# Preclinical Evaluation of a Near-Infrared Labelled Antibody Targeting the Tumour Associated Xenoantigen N-Glycolyl-Neuraminic Acid GM3 Ganglioside

**DOI:** 10.1007/s11307-025-02026-z

**Published:** 2025-06-17

**Authors:** Kris Barreto, Wendy Bernhard, Darien Toledo, Kimberly Jett, Angel Casaco, Kalet León, C. Ronald Geyer

**Affiliations:** 1 Department of Pathology and Laboratory Medicine, University of Saskatchewan, Saskatoon, SK S7N 5E5 Canada; 2 Center of Molecular Immunology (CIM), Havana, Cuba

**Keywords:** Theranostic, 14F7, NIR—near infrared Fluorophore, Molecular-targeted imaging, 14F7hT, N-glycolyl-neuraminic acid GM3 ganglioside

## Abstract

**Purpose:**

Targeted and broadly applicable molecular targets are important for image guided surgery. Xenoantigens represent a particularly interesting class of targets. This study evaluates the xenoantigen N-glycolyl-neuraminic acid GM3 ganglioside (Neu5Gc-GM3) as a potential fluorescence-guided surgical tool.

**Procedures:**

The antibody 14F7hT is conjugated to the near-infrared dye (IRDye800CW) and characterized under GLP conditions. The quality and stability of the 14F7hT-IRDye800CW probe was assessed. In vivo imaging using 14F7hT-IRDye800CW in mice with Neu5Gc GM3 positive and negative xenografts were compared to a control IgG-IRDye800CW probe targeting an epitope not present on the xenografts. Biodistribution, pharmacokinetics, and toxicity were evaluated.

**Results:**

The 14F7hT-IRDye800CW probe was 98 ± 2% pure as determined by micro-capillary electrophoresis. The KDapp as determined by binding cell-lines expressing the target was unchanged after conjugation. We demonstrate a peak accumulation window of 12 – 48 h in murine xenografts with male and female CD-1 nude mice administered 0.5 nmoles of the probe (i.v.) and very low uptake in other tissues. Preclinical toxicity studies in male and female balb/c mice support a no observed adverse effect level (NOAEL) of 50 mg/kg in mice.

**Conclusions:**

The 14F7hT-IRDye800CW probe was found to be safe and have low non-specific uptake in a model organism known to express the target. These data support future clinical development of the probe.

**Supplementary Information:**

The online version contains supplementary material available at 10.1007/s11307-025-02026-z.

## Introduction

**Fig. 1:**
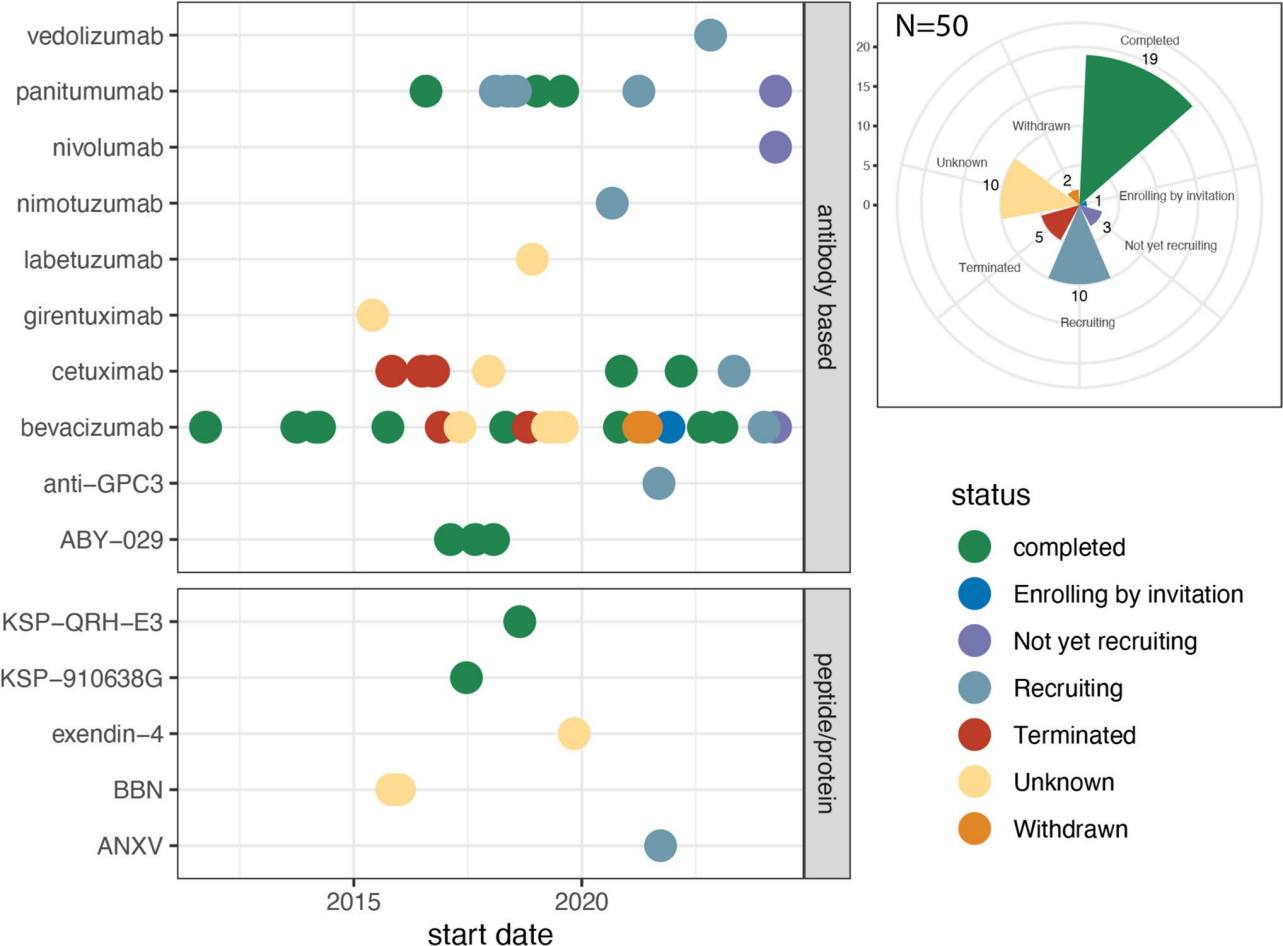
Survey of clinical trials using IRDye800CW. In the left panel, dots represent clinical trials for the indicated probe. The color indicates and position indicates the status and start date of that trial. In the upper right box data is summarized by the number of trials in each status. Data is sourced from clinicaltrials.gov (July 29, 2024)

Molecular-targeted probes can be used in fluorescence-guided surgery to assist in assessment of tumor margins and detection of sentinel lymph nodes. Currently approved fluorescent-guided surgery drugs include indocyanine green (ICG), 5-aminolevulinic Acid (5-ALA) for brain cancer, hexaminolevulinate (Cysview®) for bladder cancer, pafolacianine (Cytalux®) for ovarian cancer, and pegulicianine (Lumisight®) for breast cancer. Clinical trials in fluorescent-guided surgery commonly use near-infrared (NIR) dyes, including IRDye800CW, used in this study. Fluorescent-guided surgery is a growing area of research, with 50 clinical trials enrolling 1,097 participants using IRDye800CW probes reported on clinicaltrials.gov as of July 29, 2024 (Fig. 1). IRDye800CW has been conjugated to ten IgG-like and five peptides/protein drugs, 28% (14/50) of these trials are currently active.

Typically, molecular-targeted probes are generated against proteins or tumor-associated antigens (TAA), which are overexpressed in tumors, but are at endogenous levels in normal tissues. A subset of probes are designed to target mutant proteins unique to tumors (tumor-specific antigens (TSA)) [1]. Consequently, this requires the generation of patient-specific targeted probes [2], which is prohibitively expensive and logistically challenging.

In addition to proteins, altered glycosylation and sialylation occur in cancer cells [3–5], providing a source of TAAs as targets for developing therapeutics, referred to as tumor-associated carbohydrate antigens (TACAs). GD2 is an example of a TACA disaloganglioside that is targeted in tumors of neuroectodermal origin by therapeutic antibodies dinutuximab (Qarziba®; Unituxin®) and naxitamab (Danyelza®). Other antigens that are not produced naturally in humans and are enriched in tumors are referred to as tumor-associated xenoantigens (TAX). Many of these antigens have been identified from human transplant experiments and include Galα1-3Galβ1-4GlcNAc (alpha-Gal) and N-glycolyl-neuraminic acid (Neu5Gc) [6].

Neu5Gc-GM3 ganglioside is a sialic TAX [7]. N-acetyl-neuramic acid (Neu5Ac) and Neu5Gc are found in many species of the deuterostome lineage [8]. Notably, Neu5Gc is absent in humans and sauropsids and possibly in monotremes, such as the platypus [9]. In humans, there is a mutation initially thought to introduce a frameshift but later identified as resulting in truncation of the cytidine monophospho-N-acetylneuraminic acid hydroxylase (CMAH) enzyme responsible for converting Neu5Ac to Neu5Gc [10, 11]. Despite humans not being able to synthesize Neu5Gc, gangliosides bearing Neu5Gc are detected in a wide range of human cancers including breast cancer [12], sarcomas [13], retinoblastoma [14], non-small cell lung cancer (NSCLC) [15, 16], colon cancer [17], lymphomas [18], neuroblastoma [19], CNS tumors of various origin [20], oral melanoma [21], skin neoplasms [22], tumors of the digestive system [23], and Wilms tumors [24]. The presence of Neu5Gc GM3 on cancer cells is attributed to diet incorporation (red meat or milk products) [25], due to increased micropinocytosis [26], higher metabolic rate [27], and induction of the sialic acid transporters by hypoxia [28, 29].

To construct a molecular-targeted probe against Neu5Gc-GM3, a highly specific antibody is needed. An anti-Neu5Gc-GM3 monoclonal antibody (14F7hT) has been developed, which has high specificity for Neu5Gc-GM3 and does not react with the Neu5Ac-GM3 counterpart or other related gangliosides. 14F7 was first isolated as a murine IgG1-kappa antibody [30]. Crystal structures of the Fab (1R1H) [31] and scFv (6FFJ, 6S2I) have been solved [32, 33]. A humanized version (14F7hT) has been produced (GenBank: AY331718.1/AY331717.2) [34], which shows antibody-dependent cellular cytotoxicity (ADCC) [35, 36]. The single-chain variable fragment of 14F7hT has been used to construct a chimeric antigen receptor T-cell [37, 38].

Neu5Gc-GM3 has been detected by immunohistochemistry using 14F7 in many human cancers [12–24]. 14F7 staining in tumors is a negative prognostic marker indicating poor patient outcome [13, 15, 17, 39]. The clinical value of Neu5Gc-GM3 as a therapeutic target for cancers has also been demonstrated through the successful approval of an anti-idiotypic Neu5Gc vaccine, racotumomab (Vaxira) for lung cancer (https://www.vaxira.com/en_index.html) and a Neu5Gc-GM3 vaccine, GlycoVaxGM3, in breast cancer [40].

Here, we characterized the pre-clinical imaging properties of 14F7hT conjugated to the near-infrared fluorescent dye IRDye800CW (14F7hT-IRDye800CW) with Neu5Gc-GM3 positive cell lines in a murine xenograft model. We determined GMP specifications for 14F7hT-IRDye800CW production and characterized its biodistribution, pharmacokinetics, and toxicity in mice.

## Materials and Methods

### Quality Control

Antibody conjugation and quality control are provided in the Supplementary Materials. The final IRDye800CW-conjugated antibody was characterized for visual appearance, strength (concentration), labelling ratio (dye to protein ratio), purity, cell binding (flow cytometry), pH, and endotoxins (Supplementary Materials).

### Fluorescent Imaging and Biodistribution

For P3X63Ag8.653 xenografts, 0.5 nmoles of antibody were injected via the tail vein in a 200 µL bolus dose. Fluorescent images were taken using the LI-COR Pearl Impulse small animal imaging system. The excitation and emission settings were 785 and 820 nm, respectively. Raw images were processed in R (The project for statistical computing) [41] for visualization, analysis was performed on raw images. Processed, images were imported using a custom script and smoothed using a median pixel area of 4 using the EBimage package [42], normalized by dividing each pixel by the labelling ratio, and plotted using the ggplot2 package [43]. Raw images were quantified using Image Studio Software (version 3.1).

### Pharmacokinetics

Female Balb/c mice (*n* = 9) were injected with 0.5 nmoles of 14F7-IRDye800CW via the tail vein (75 µg probe/20 g mouse). PK analysis was performed in R using the PK package [44].

### Acute and Delayed Toxicity

A single 200 µL dose of 1 mg (6.7 nmoles) IRDye800CW-14F7hT or vehicle (PBS) was injected via the tail vein in 7–9 week old BALB/c mice weighing 20 ± 2 g (18 ± 1 g females, 21 ± 1 g males) to determine acute (day 2) and delayed (day 14) toxicity. All animals in this study were observed regularly for signs of mortality, morbidity, injury, and intake of food and water. Mice were monitored for weight loss and daily clinical observation. Forty-eight mice (24 male + 24 female) were divided into groups (Supplementary Fig. 5; Supplementary Materials). Statistical analysis was nonparametric and predetermined, we used GraphPad prism version 9, using Krusal-Wallis test followed by Dunn’s multiple comparisons test. Comparisons were made between treatment and baseline or treatment and untreated for each group. Adjusted *p*-values are reported. Plots were generated in R (The project for statistical computing) [41] using ggplot2 [43].

## Results

### Interaction of 14F7hT with Cell-Lines

**Fig. 2:**
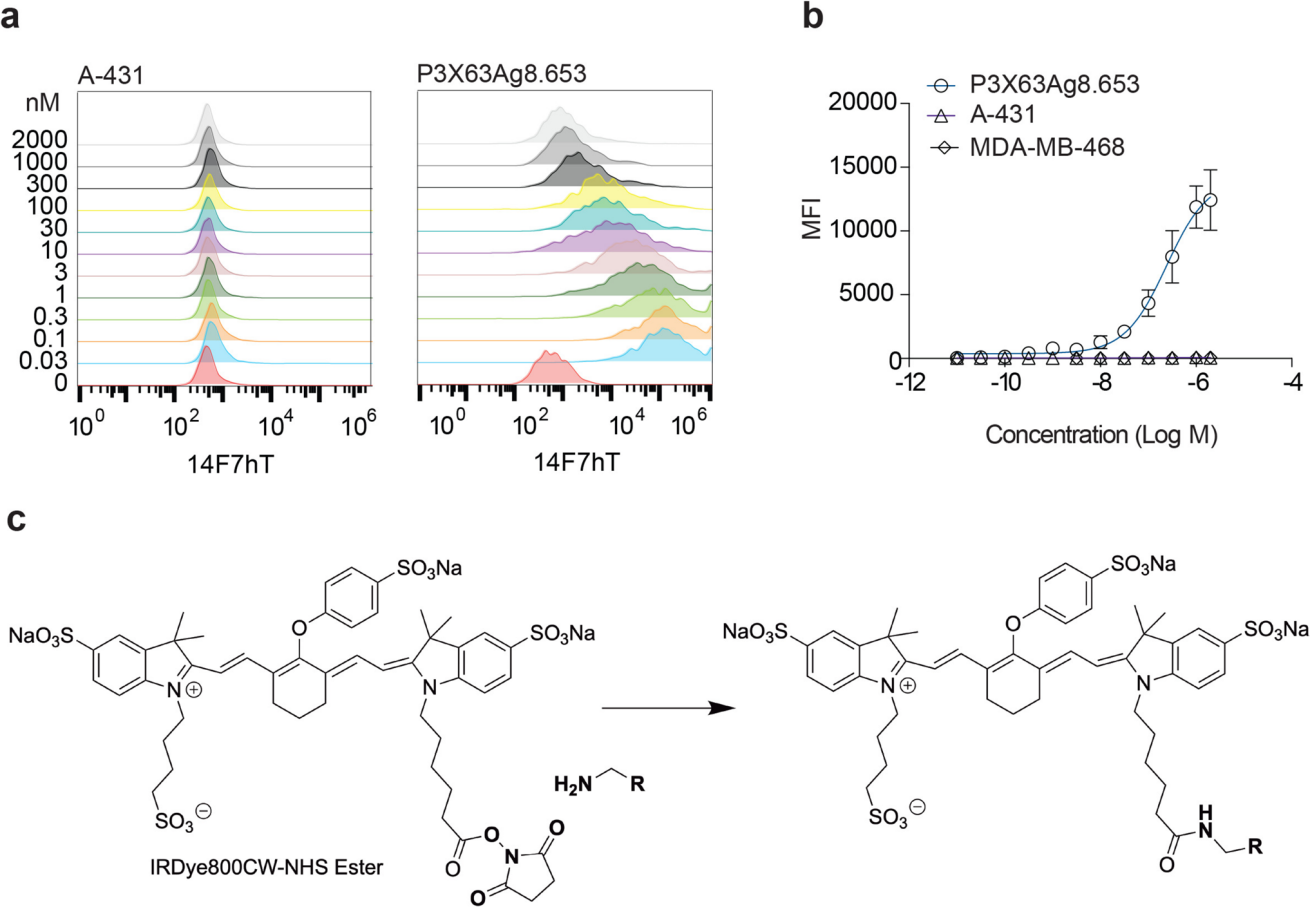
Analysis of 14F7hT binding to cell lines. **a** Flow cytometry analysis of 14F7hT binding to human A-431 and murine P3X63Ag8.653 cell lines, which are Neu5Gc GM3 negative and positive, respectively. Concentrations of 14F7hT are shown on the y-axis in nM. **b** Binding curves of mean fluorescent intensity (MFI) versus concentration of 14F7hT for P3X63Ag8.653, A-431, and MDA-MB-486 (Neu5Gc-GM3 negative). **c** Schematic of conjugation of IRDye800CW-NHS ester to primary amines on the antibody 14F7hT (**R**) resulting in the formation of an amide bond.

We tested the ability of 14F7hT to bind cell lines using flow cytometry (materials and methods are in Supplementary Materials, Fig. 2). 14F7hT bound the Neu5Gc-GM3 positive murine cell line P3X63Ag8.653 [36], resulting in a broad histogram (Fig. 2a). At concentrations as low as 1 nM, a tenfold increase in mean fluorescent intensity (MFI) was observed, which saturated at 140,000 ± 20,000 MFI (Fig. 2b). The apparent dissociation constant (KD,app) calculated from the titration curve was 240 ± 20 nM. The human cell lines A-431 and MDA-MB-468 were negative for 14F7hT staining and showed a MFImax < 1000 MFI.

### Preparation and Characterization of 14F7hT-IRDye800CW

We conjugated NHS-IRDye800CW to 14F7hT at a dye:protein labeling ration of ~ 1. The NHS group reacts with a primary amine on the antibody forming a stable amide bond (Fig. 2c). 14F7hT-IRDye800CW was prepared under good laboratory practice (GLP) conditions with a labeling ratio of 1.2 ± 0.2. The observed molecular weight of conjugated versus unconjugated 14F7hT was 151 ± 4 kDa and 152 ± 4 kDa, respectively. The apparent dissociation constant of 14F7hT-IRDye800CW changed less than twofold relative to 14F7hT. The proposed clinical trial quality release specifications for 14F7hT-IRDye800CW are shown in Supplementary Table 1.

14F7hT-IRDye800CW remained within specification during short term storage at 4 °C or room temperature (18—25 °C). The purity of 14F7hT-IRDye800CW after 18 months at −80 °C was unchanged from end of synthesis (97%). The fluorescence purity assessed was 98%. The functional activity is within specification at 62%. The 95% confidence interval (CI) for strength goes out of specification at 10 months. All other tested parameters are within specification up to 10 months. Therefore, these data support a 10-month shelf-life for 14F7hT-IRDye800CW (Supplementary Figs. 1, 2) [45, 46]. The KD,app ratio was not used to determine shelf-life because the 6-month datapoint was not collected, which contributes to the large 95% CI.

### In Vivo Imaging with the 14F7hT-IRDye800CW Probe Using a Murine P3X63Ag8.653 Xenograft Model

**Fig. 3:**
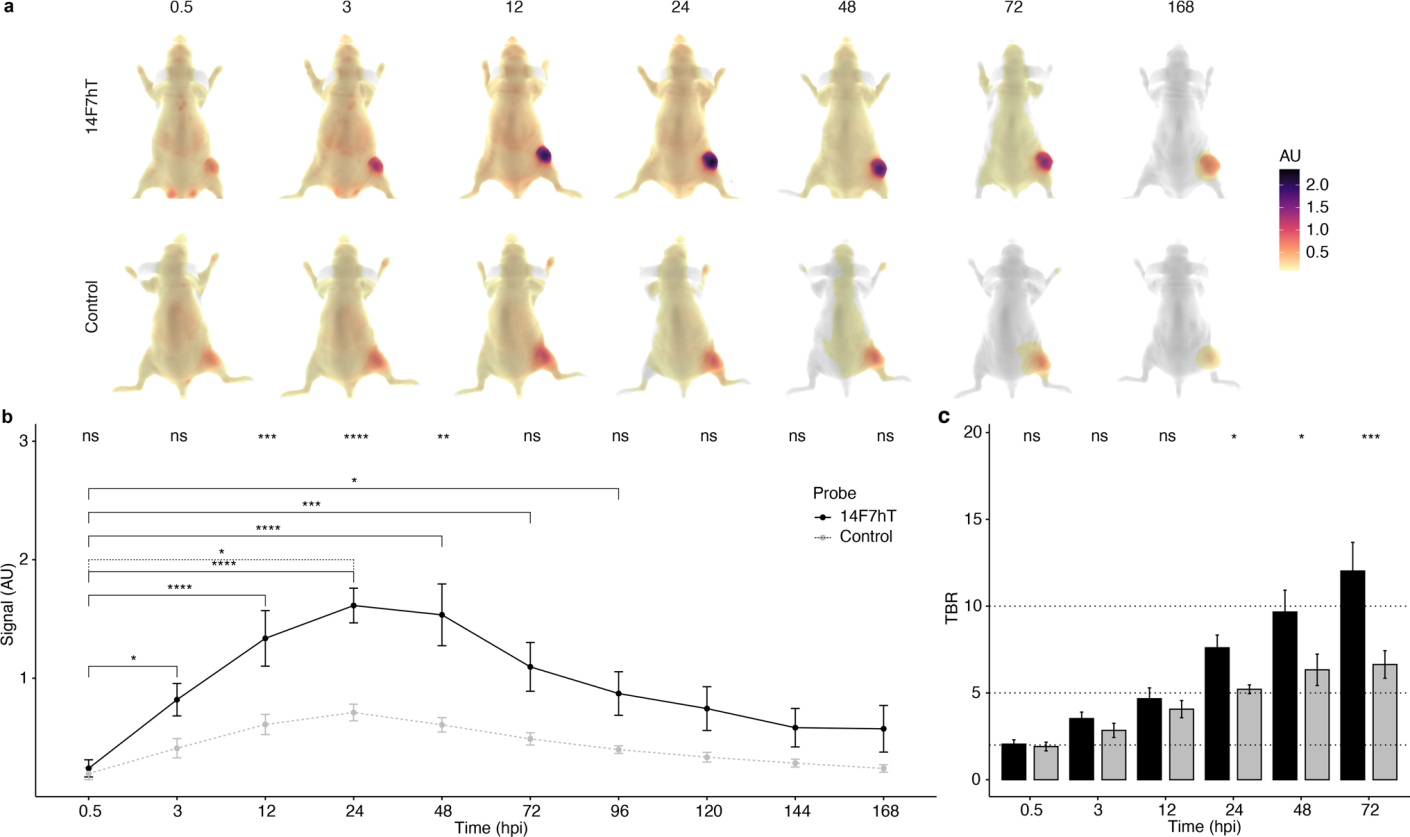
Fluorescent imaging in P3X63Ag8.653 xenografts. CD-1 nude mice bearing P3X63Ag8.653 xenografts were injected with either 14F7hT-IRDye800CW or a control IgG-IRDye800CW. **a** Dorsal fluorescent images (arbitrary units) of mice at indicated hours post injection (hpi). **b** Quantification of the fluorescent signal in images. **c** Tumor-to-background ratio (TBR) analysis of images. Data is presented as mean ± sem. Significance is tested from time zero within each treatment (bars, solid: 14F7hT, dotted: IgG), and between treatments (IgG vs 14F7hT; top).

We evaluated the in vivo imaging properties of 14F7hT-IRDye800CW using the murine lymphoblast P3X63Ag8.653 cell line. Ten mice (5 male and 5 female for each probe, total = 20), were imaged (Fig. 3). Mice were sacrificed at days 1 and 7 when organs were removed and imaged.

14F7hT-IRDye800CW accumulated at higher levels in the P3X63Ag8.653 xenograft relative to the control IgG-IRDye800CW as shown in the dorsal images (Fig. 3). 14F7hT-IRDye800CW accumulated mainly in the xenograft and primarily cleared through the liver with no significant (*p* > 0.5) difference between the two probes at any time-point (Supplementary Fig. 2). Kidney clearance was not significantly different from the background in the forearm of the mouse (Supplementary Fig. 2B). The fluorescent signal in the P3X63Ag8.653 xenograft of mice injected with 14F7hT-IRDye800CW peaked between 12 and 48 h. There were significant differences in the fluorescent signal between the initial time-point (0.5 h) and 3–96 h post injection (hpi). The control only showed a significant (*p* < = 0.05, dotted line) increase in fluorescence signal over the initial time-point at 24 h (Fig. 3). Mice injected with 14F7hT-IRDye800CW had significantly more signal in xenografts than mice injected with the control at 12, 24, and 48 h (Fig. 3). The tumor-to-background ratio (TBR) was significantly different between control and 14F7hT-IRDye800CW probes at 24 (*p* < = 0.05), 48 (*p* < = 0.05), and 72 h (*p* < = 0.01) (Fig. 3). The TBR for 14F7hT-IRDye800CW was 8 ± 2 at 24 h and increased to 10 ± 3 by 48 h, indicating an optimal imaging time between 24–48 h, where both the signal and TBR are high and significantly different from the control.

### Biodistribution of 14F7hT-IRDye800CW in Murine P3X63Ag8.653 Xenograft Model

**Fig. 4:**
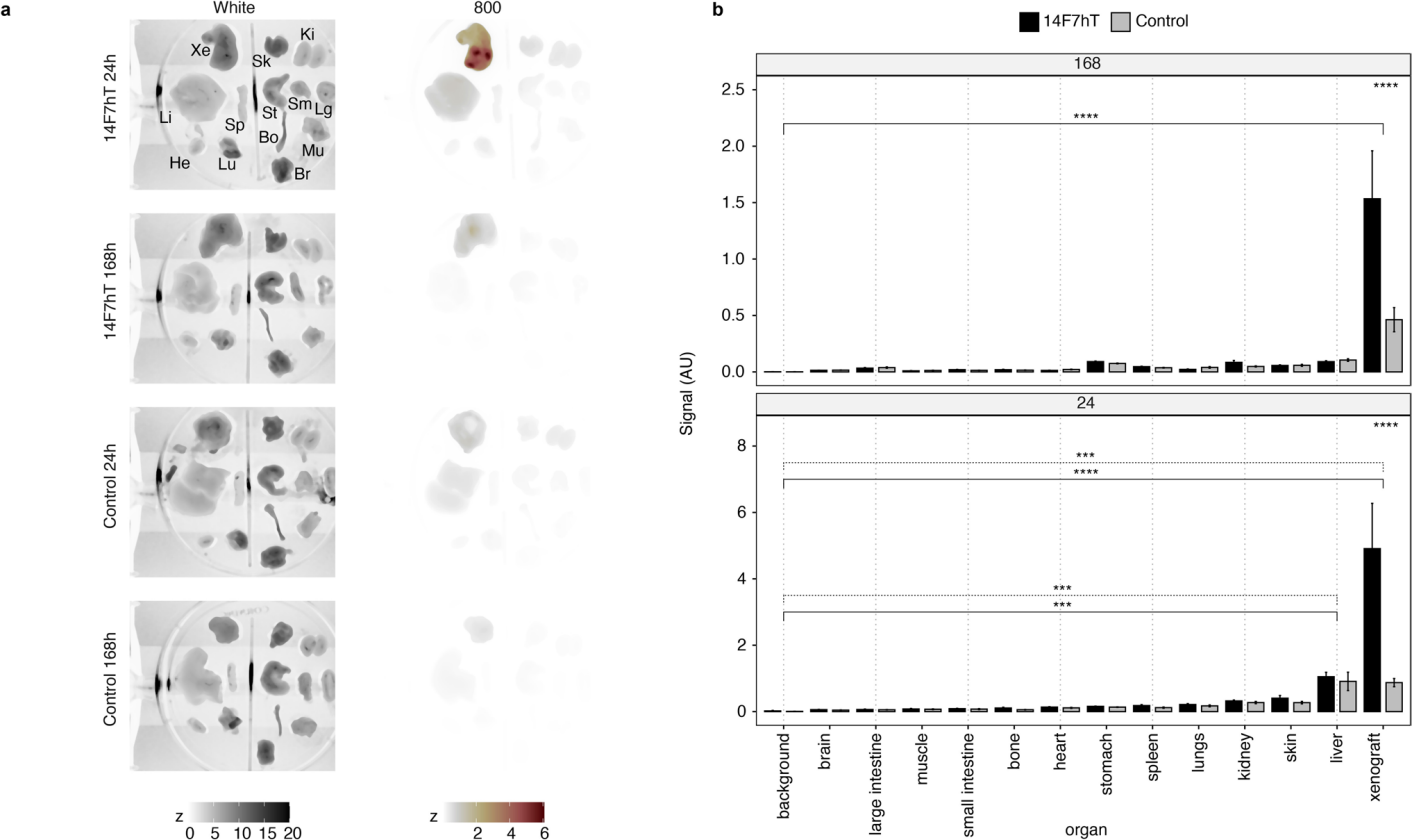
Biodistribution of 14F7hT and control IgG-IRDye800CW probes in P3X63Ag8.653 CD-1 nude xenografted mice. **a** Representative images of organs ex vivo. White light and 800 nm channel images of scanned organs. Organs from left to right starting from the top row are: Row 1: xenograft (Xe), skin (Sk), kidney (Ki), Row 2: liver (Li), spleen (Sp), stomach (St), small intestine (Sm), large intestine (Lg), Row 3: heart (He), lungs (Lu), bones (Bo), muscle (Mu), Bottom: and brain (Br). Z is pixel intensity. **b** Quantitative analysis of fluorescence in organs ex vivo. Data is presented as mean ± sem.

Ex vivo biodistribution of 14F7hT-IRDye800CW and a control were analyzed in the P3X63Ag8.653 xenograft model at 24 and 168 h (Fig. 4). The 14F7hT-IRDye800CW probe primarily cleared through the liver. The xenograft had ~ fivefold higher signal than the liver (Fig. 4). The only tissues with significantly more signal than background at 24 hpi for 14F7hT-IRDye800CW were the xenograft (*p* < = 0.001) and liver (*p* < = 0.05). By 7 days, the signal in the xenograft was the only organ to have significantly more signal than background, indicating clearance from all other tissues, including the liver.

The control cleared primarily through the liver and showed some early uptake in the xenograft, which cleared by 7 days. There were significant differences at 24 hpi between background and xenograft (*p* < = 0.001) and liver (*p* < = 0.001). By 168 hpi there were no significant differences between the xenograft and background (*p* > 0.05).

The only significant differences between 14F7hT-IRDye800CW and the control in the biodistribution study were in the xenograft. There was significantly higher uptake at day 1 (5 ± 1 vs 0.9 ± 1 SEM) (*p* < = 0.0001) and day 7 (*p* < = 0.0001). No other organs had significant differences in uptake (Fig. 4b).

### Characterization of 14F7hT-IRDye800CW Binding in the A431 Neu5Gc-GM3 Negative Cell Line Xenograft

**Fig. 5:**
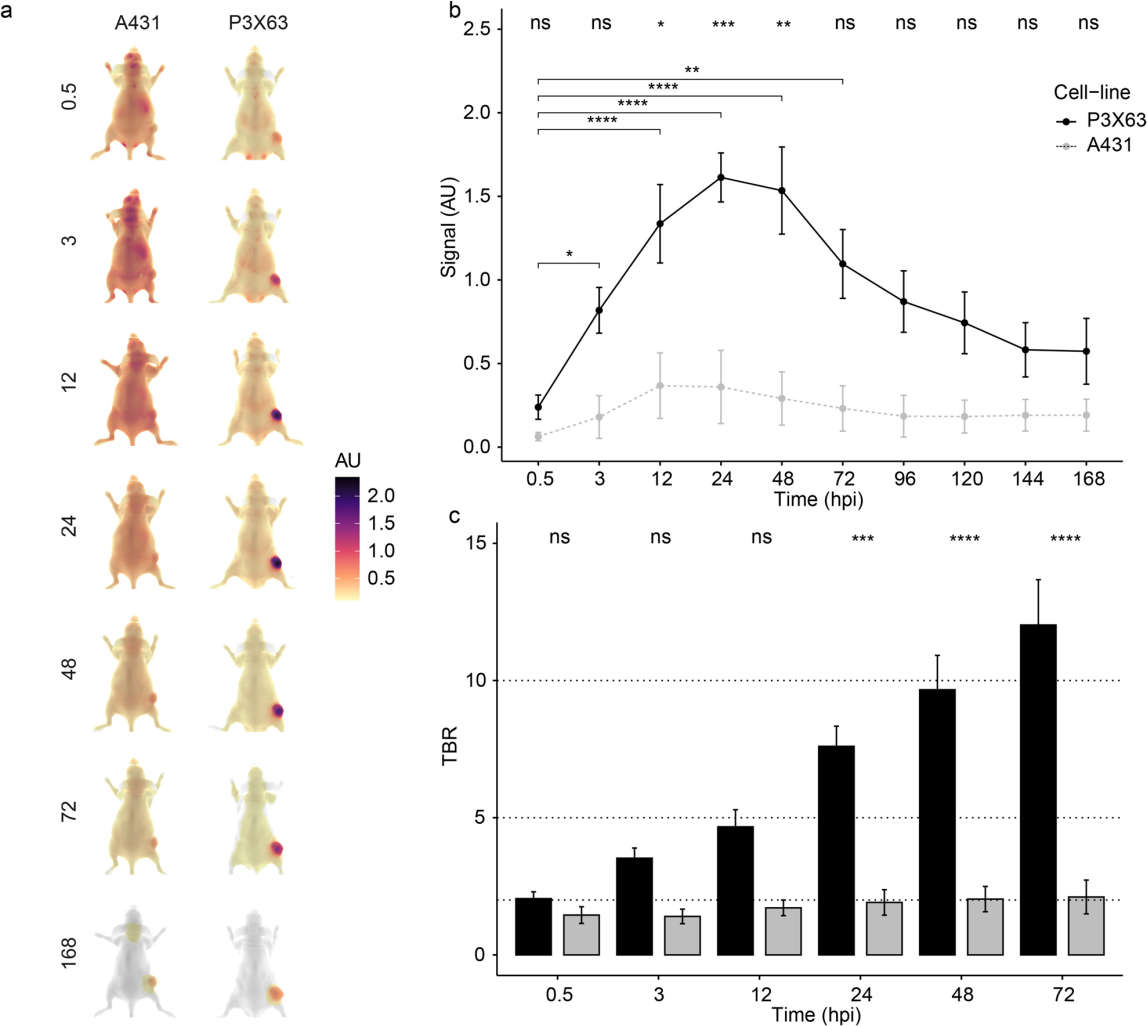
Comparison of mice bearing Neu5Gc-GM3 positive (P3X63 = P3X63Ag8.653) and negative (A431 = A-431) xenografts injected with 14F7hT-IRDye800CW. **a** Dorsal fluorescent images at indicated time-points (hpi). **b** Analysis of fluorescent signal in xenografts from the mice images. **c** Tumor-to-background (TBR) ratios of mouse images.

To further characterize the specificity of the 14F7hT-IRDye800CW probe mice bearing A-431 xenografts were imaged (Fig. 5). The probe primarily cleared through the liver (Supplementary Fig. 3B). A-431 xenografts showed low levels of uptake (MFI < 0.5), which was similar to the control and the signal peaked slightly earlier between 12 and 24 hpi. The P3X63Ag8.653 xenografts had significantly higher signal (~ fourfold) at 24 h than Neu5Gc-GM3-negative xenografts and peaked slightly later between 24 and 48 hpi. Tumor to background ratios (TBR) at 48 h for 14F7hT-IRDye800CW probe with P3X63Ag8.653 xenografts were significantly higher (10 ± 3) compared to TBR of 2 ± 1 for A-431 xenografts.

**Fig. 6:**
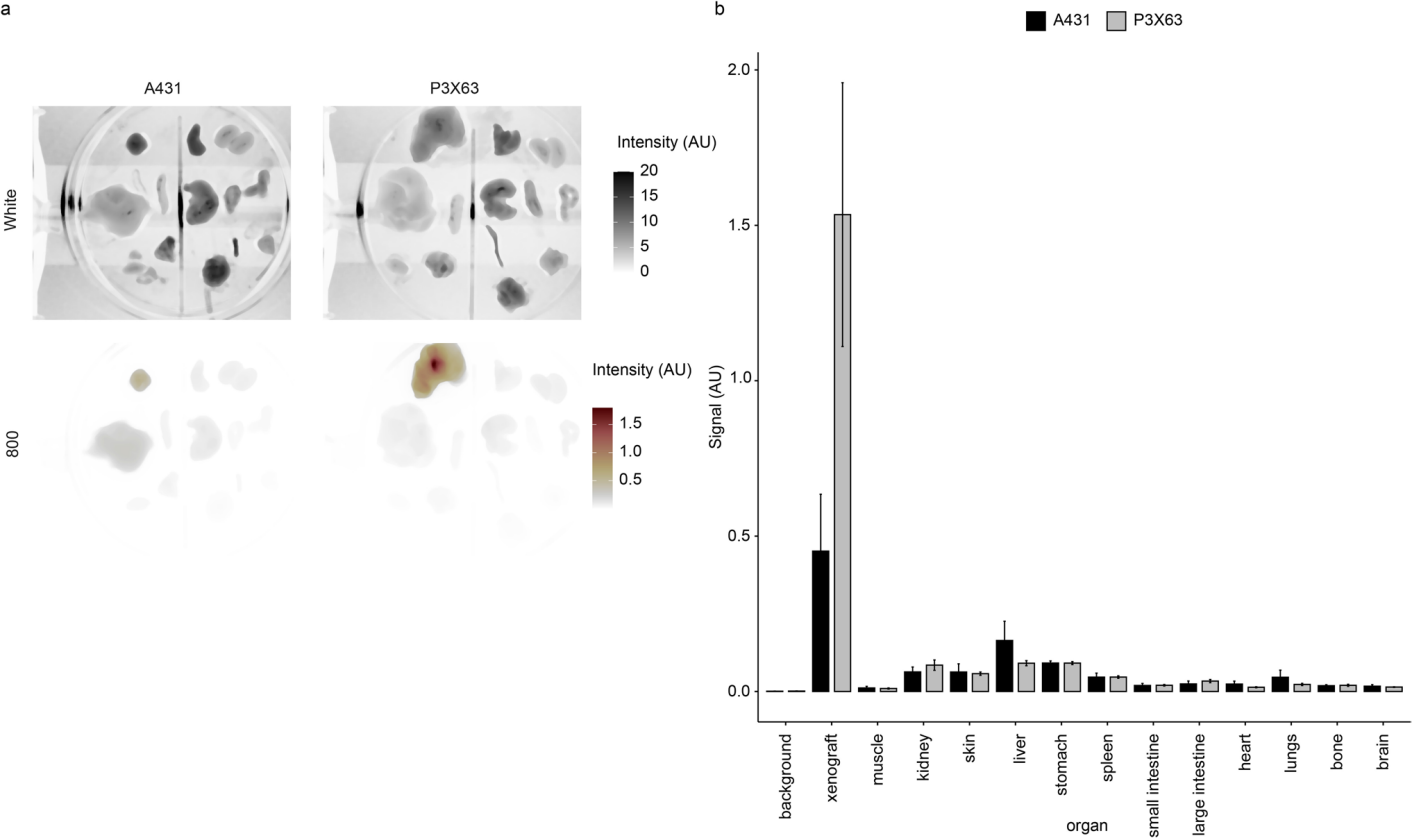
Biodistribution of 14F7hT-IRDye800CW injected into CD-1 nude mice bearing Neu5GC-GM3 positive or negative xenografts. **a** Ex vivo images of organs under white light and the 800 nm channel. Organs from left to right starting from the top. Row 1: xenograft, skin, kidney, Row 2: liver, spleen, stomach, small intestine, large intestine, Row 3: heart, lungs, bones, muscle, Bottom: and brain. **b** Quantitative fluorescence analysis of ex vivo images. Fluorescence signal is reported as MFI per pixel. Data is presented as mean ± sem.

Mice were sacrificed after 7 days and organs were removed and imaged (Fig. 6). Significantly more signal was observed in P3X63Ag8.653 xenografts (~ 1.5 AU) than A-431 xenografts (< 0.5 AU). By 7 days, the probe had cleared from most tissues with liver, stomach and kidney having the highest uptake (Fig. 6c).

### Pharmacokinetics and Toxicity of 14F7hT-IRDye800CW

Pharmacokinetics were determined by non-compartmental analysis to have a half-life of 1.13 ± 0.03 days, a clearance of 70 ± 5 ml day^−1^ kg^−1^, and volume of distribution at steady state of 116 ± 8 mL kg^−1^ (Supplementary Fig. 4). Two compartmental analysis gave similar results, exhibiting a bi-phasic half-life with a fast distribution t_1/2,_$\alpha$ of 0.5 h and elimination of t_1/2,_$\beta$ of 1.16 days (Supplementary Fig. 4).

Imaging probes are administered as a single injection, thus a single dose acute and delayed toxicity in normal balb/c mice was used to determine toxicity. The median mouse weight at the time of injection was 20 ± 2 g (*n* = 48), corresponding to a 50 mg/kg dose, or a 3 g dose in a 60 kg human. This represents a 4.1 mg/kg human equivalent dose (HED) based on surface area [47], or a 246 mg dose in a 60 kg human.

Mice (*n* = 48) were divided into two cohorts (Supplementary Fig. 5). All mice in the study survived until their scheduled necropsies. No remarkable affects were observed. One mouse in the baseline group developed a cyst on the right kidney, so the weight of the left kidney was used in the analysis for this mouse. There was no significant difference in the body weight or organ weights between treated and vehicle groups (Supplementary Fig. 6). The clinical chemistry parameters evaluated are listed in Supplementary Table 2 and the resulting measurements are shown in Supplementary Tables 3, 4 and Supplementary Figs. 7, 8. No significant differences were observed for any parameters between baseline vs treatment and vehicle vs treatment. Hematology parameters analyzed are listed in Supplementary Table 5 and results are provided in Supplementary Tables 6, 7 and Supplementary Fig. 9. There were no significant differences between either treatment vs vehicle and treatment vs baseline groups for any of the parameters.

These data support a no observed adverse events level (NOAEL) of a human equivalent dose (HED) of 4.1 mg/kg based on surface area or 50 mg/kg by equivalent mass dose.

### Analysis of Neu5Gc-GM3 Levels in Human Cancers

14F7 has been used to detect Neu5Gc-GM3 in a wide range of human cancers [12–24]. Analysis of aggregate data from these 13 studies shows 81.2% (580/714) of tissue of any tumor type were associated with 14F7 staining but only 4.9% (8/164) of normal tissues. Of these normal tissues that stained with 14F7, 2.4% (4/164) were found in abnormal or benign tissues the remaining 2.4% (4/164) were from gastrointestinal tissues (Supplementary Methods).

## Discussion

14F7hT-IRDye800CW showed excellent tumor accumulation and a high tumor to background ratio of 8 ± 2 after 24 hpi. The maximum signal in the tumors was reached at 24 hpi, which was significantly earlier than that observed with other antibody imaging probes, such as 48 hpi for cetuximab-IRDye800CW [48, 49], 72 hpi for panitumab-IRDye800CW [48], and 96 hpi for nimotuzumab-IRDye800CW [50].

The half-life of 0.5 nmoles of 14F7hT-IRDye800CW administered i.v. was 1.13 ± 0.03 days, which is shorter than anti-EGFR IgG nimotuzumab-IRDye800CW 1.58 ± 0.06 days [51], but still clears slower than smaller antibody fragments such as anti-EpCAM Fab-IRDye800CW with a half-life of 0.19 days [52].

The interaction of 14F7hT-IRDye800CW with endogenous Neu5Gc-GM3 in mice was surprisingly low given that mice have a functional CMAH gene and synthesize Neu5Gc-GM3 [36]. In murine biodistribution studies, normal tissues had no significant uptake of the 14F7hT probe compared to the control nimotuzumab probe, except for the liver, which is the main route of clearance for IgGs. The apparent dissociation constant of 14F7hT-IRDye800CW for P3X63Ag8.653 was relatively weak (240 ± 20 nM), which may be why it accumulated in xenografts with high levels of Neu5Gc-GM3 and not in tissues having endogenous levels of Neu5Gc-GM3. “Affinity optimized” antibody binding, where an antibody has relatively weak binding to its target has been suggested previously to explain why nimotuzumab has low adverse reactions compared to other anti-EGFR antibodies as the weaker binding ensures it only accumulates on cells with high levels of expression [53]. Similarly, 14F7-99mTc was used in a human breast cancer SPECT imaging clinical trial and resulted in no false positives in 13 participants [12]. These results suggest that accumulation of 14F7hT will be low in normal human tissues, which is consistent with studies showing that Neu5Gc-GM3 is present in tumors and rarely detected in corresponding normal tissues.

Studies have linked the accumulation of Neu5Gc in human tissues to diet [25]. Aggregate data analysis from human tissues showed that 4.9% (8/164) of normal tissues exhibited 14F7 accumulation. Half of these (2.4%) were from gastrointestinal tissues, likely due to their proximity to dietary supply routes. This finding is consistent with studies in CMAH-/- knockout mice, which show intestinal processing of Neu5Gc-glycoproteins [54]. Similarly, studies where humans were fed a Neu5Gc diet showed incorporation into glycoproteins [55]. Other studies have suggested that accumulation in cancer tissues is due to increased macropinocytosis [26], higher metabolic rates [27], and the induction of transporters due to hypoxia [28, 29]. Understanding the Neu5Gc target is complicated by its context, whether displayed as a glycolipid or glycoprotein [56]. 14F7 is highly specific for the ganglioside Neu5Gc-GM3 and requires the sphingolipid part for recognition [57]. In contrast, anti-Neu5Gc IgY antibodies recognize a variety of glycans modified with a terminal Neu5Gc moiety [58]. 14F7 has properties that make it useful as a theranostic as it recognizes Neu5Gc-GM3, which is more tumour specific.

Although statistical differences were observed in the toxicology study, the statistical plan was designed to increase sensitivity by decreasing the number of groups in each statistical analysis at the risk of increasing false positives. This was mitigated by including two control groups (baseline and vehicle) and classifying only a statistical difference between both baseline vs treatment and vehicle vs treatment as an event. In the clinical chemistry parameters three markers of kidney function (anion gap, phosphorus and urea) were found in females. In males, differences included markers of liver function GLDH and globulin. Globulin is indirectly measured by total protein which measures both albumin and globulin, which was also different in males. In the complete blood count (CBC) parameters only RBC and HGB were different in males at 2 days however no differences were seen between vehicle and treatment groups, and values in the treatment group were within normal ranges [59].

There were no trends between males and females, any differences were small, and no values were outside of normal ranges. The number of statistical differences detected are consistent with false discovery at an alpha of 0.05 as > 100 statistical tests were performed. Most importantly no differences between both baseline vs treatment and vehicle vs treatment indicates there are no safety concerns.

According to guidance documents from the FDA the maximum recommended starting dose (MRSD) for first-in-human clinical trials is determined by the no observed adverse effect level (NOAEL) in a relevant animal model with a default safety factor of 10 [60]. The safety factor is based on the possibility that humans may be more sensitive to the toxic effects of a therapeutic than animal models. In this case, an argument could be made that the animal model is likely to be more sensitive as mice express the CMAH gene whereas humans do not. Previous clinical experience with 14F7hT may also justify lowering this safety factor. 14F7hT has been used in human trials [61], and previous clinical trials with IRDye800CW have indicated there is no additional safety risk from conjugation with IRDye800CW [51], which could also justify using a lower safety factor. The no observed adverse effect level in this study was 50 mg/kg. No significant differences were observed in organ and body weights, clinical chemistry, or hematology parameters. No abnormal behavior was observed in either group. This represents a human equivalent dose (HED) by mass of 3000 mg/participant and a 250 mg/participant by surface area calculated according to FDA guidance document [60]. Proteins administered intravascularly with Mr > 100,000 daltons should be normalized to mg/kg according to guidance documents [60, 62]. Antibodies have weights of 150,000 daltons. Applying the default safety factor of 10 and assuming a 60 kg participant, results in a maximum recommended starting dose of 25 mg/participant by surface area or 300 mg/participant by mass. Given that the route of administration would be intravenously, normalization by mass giving a MRSD of 300 mg/participant would be appropriate for first-in-human trials. This starting dose is more than what is typically administered in fluorescent-guided surgery trials (< = 100 mg/participant).

## Conclusions

In summary, 14F7hT-IRDye800CW is a good candidate antibody for fluorescent-guided surgery applications. It shows high tumor accumulation and high tumor to background ratio at 24 h. 14F7 labeled with 99mTc was used in a clinical trial [12] and showed uptake in breast cancer tumors. The favorable imaging properties observed in mice where endogenous Neu5Gc-GM3 is present at low levels, suggest that 14F7hT will perform well in humans where Neu5Gc needs to be acquired from the diet and is preferentially incorporated in tumor GM3 gangliosides.

## Supplementary Information

Below is the link to the electronic supplementary material.

Supplementary file1 (DOCX 7.67 MB)

## Data Availability

All data generated or analyzed during this study are included in this published article and its supplementary information files.
