# Supplementary material for: Preclinical Evaluation of a Near-Infrared Labelled Antibody Targeting the Tumour Associated Xenoantigen N-Glycolyl-Neuraminic Acid GM3 Ganglioside
Source: Mol Imaging Biol. 2025 Jun 17;27(4):518–28. doi: 10.1007/s11307-025-02026-z (PMC12405320; doi:10.1007/s11307-025-02026-z)
Supplement: Supplementary file 1 — Supplementary file1 (DOCX 7.67 MB) [file 11307_2025_2026_MOESM1_ESM.docx]

*Preclinical evaluation of a near-infrared labelled antibody targeting the tumour associated xenoantigen N-glycolyl-neuraminic acid GM3 ganglioside.*

Kris Barreto^1^, Wendy Bernhard^1^, Darien Toledo^2^, Kimberly Jett^1^, Angel Casaco^2^, Kalet León^2^, C. Ronald Geyer^1^

1Department of Pathology and Laboratory Medicine, University of Saskatchewan, Saskatoon, SK, S7N 5E5, Canada
2Center of Molecular Immunology (CIM), Havana, Cuba

Corresponding Author: C. Ronald Geyer [clg595@mail.usask.ca](mailto:clg595@mail.usask.ca), 306-966-2040

Shortened Title: Preclinical Evaluation of NIR-Labeled Antibody Targeting Tumor-Associated Xenoantigen Neu5Gc GM3

Manuscript Category: Research Article

Supplementary materials

*Cell lines and cell culture*

P3X63Ag8.653 (CRL-1580) and A-431 (CRL-1555) cell lines were obtained from ATCC and cultured at 37°C with 5% CO_2_ in 90% Roswell Park Memorial Institute medium (RPMI) + 10% FBS. MDA-MB-468 (HTB-132) were obtained from ATCC and cultured at 37°C with 5% CO_2_ in 90% Dulbecco’s modified Eagles medium (DMEM) + 10% FBS. P3X63Ag8.653 (CRL-1580) were purchased for this project from ATCC. A-431 were authenticated by The Centre for Applied Genomics, Toronto, Ontario (<https://www.tcag.ca/facilities/geneticAnalysis.html>).

*Antibody conjugation and Quality control*

All antibodies were kindly provided by the Center of Molecular Immunology (CIM) in Cuba and included humanized 14F7 (14F7hT; anti-GM3(Neu5Gc) ganglioside) [[*34*](#ref-PMID:21802167)] and Nimotuzumab (anti-EGFR). IgG-IRDye800CW and 14F7hT-IRDye800CW were prepared by mixing antibody (5 mg/mL) with 3-fold molar excess of IRDye800CW NHS Ester (LiCor, USA) (10 mg/mL) in PBS and incubated for 1 hour at room temperature. Unreacted dye was removed using a Zeba spin column, according to manufacturer’s instructions.

The final IRDye800CW-conjugated antibody was characterized for visual appearance, strength, labelling ratio, purity, cell binding, pH, and endotoxins.

Visual appearance: The appearance of the solution was analyzed to confirm that there was no particulate matter.
Strength: The concentration of IRDye800CW-conjugated antibody was determined by measuring the UV absorbance at 280 nm and 789 nm with a Nanodrop 2000. Baseline correction was applied at 450 nm and samples were diluted if any absorbance values were greater than 1.0. The concentration was calculated using equation 1, where E_protein_ = 203,000 M^-1^ cm^-1^, E_dye_ = 270,000 M^-1^ cm^-1^, and MW_protein_ = 150,000 Da.

Equation 1: $ProteinConcentration\left( mg/mL \right)=MW_{protein}*\frac{\left( A_{280}-\left( 0.03*A_{789} \right) \right)}{E_{protein}}$

Labelling ratio (dye:protein): The number of IRDye800CW molecules per antibody was determined by measuring the absorbance at 789 nm. The labeling ratio was calculated using equation 2, using the constants from equation 1.

Equation 2: $LabellingRatio\left( Dye:Protein \right)=\frac{\left( A_{789nm}/E_{dye} \right)}{(\left( A_{280nm}-\left( 0.03xA_{789nm} \right) \right)}/E_{protein}))$

Purity (fluorescence): The purity of IRDye800CW-conjugated antibody was determined by running a 1:1000 dilution of the IRDye800CW-conjugated antibody on a 15% pre-cast SDS-PAGE at 200 volts for 25 minutes. The gel was then scanned on a LICOR odyssey scanner using the 800 nm channel.

Purity (protein) and molecular weight (kDa): Micro-capillary electrophoresis was used to determine the molecular weight and purity of the IRDye800CW-conjugated antibody according to the manufacturer’s instructions (Agilent, Bioanalyzer 2100).

Cell binding assay: Flow cytometry was used to assess the difference in binding between IRDye800CW-14F7hT and 14F7hT. Cell-lines A-431, P3X63Ag8.653, and MDA-MB-468 were characterized for binding as described previously [[*41*](#ref-PMID:30867810)]. Briefly, 100,000 cells were washed with PBSF (phosphate buffered saline + 2% FBS) and incubated for 30 min with dilutions of 14F7hT or 14F7hT-IRDye800CW or nimotuzumab-IRDye800CW, ranging from 0-2 µM. Volumes were adjusted to ensure a 10-fold molar excess of antibody. Cells were incubated for 15 min at 4°C and then washed with PBSF to remove unbound antibody. Cells were stained at 50x dilution with a goat anti-human Fab2-FITC secondary antibody for 30 mins at 4°C. Cells were washed with PBSF to remove unbound secondary antibody and analyzed on a Beckman Coulter Gallios flow cytometer. MFI was determined using FlowJo version 10 and fit to a one-site binding curve in Prism version 6 to determine the apparent binding constant (KD,app).

pH: The pH of antibody solutions was measured using a pH meter (Orion Star™A211 (13-645-521PM)) with a micro pH probe (8220BNWP) calibrated the same day with USP standards 4.01, 7.00, and 10.01 (slope >= 95%).

Endotoxin: Endotoxin levels in the final product were measured using the Endosafe-Portable Test System (Charles River Laboratories).

Mice

All animals used were cared for and maintained under the supervision and guidelines of the University of Saskatchewan Animal Care Committee, which maintains sentinel animals. CD-1 nude mice and balb/c mice were obtained from Charles River (St-Constant, Quebec, Canada) or Charles Rover Laboratories (Hartford, CT) at 4 weeks of age and housed in a 12 h light, 12 h dark cycle in a temperature and humidity-controlled vivarium. Animals had ad libitum access to mouse diet balb/c (Lab diet, ProLab RMH 3000), CD-1 nude (Lab diet, Picolab 5053) and water. Maximum caging density was 5 mice per cage. Mice were allowed to acclimatize for a minimum of one week before handling. Mice were obtained, housed, and euthanized in accordance with University Animal Care Committee (UACC) guidelines (protocol # 20160112).

*Mouse xenografts*

Mice were obtained, housed, and euthanized in accordance with University Animal Care Committee (UACC) guidelines (protocol # 20160112). CD1-athymic nude male or female mice (Charles River Laboratories, Hartford, CT) were used for the xenograft models. 10 x 10^6^ cells were washed using growth media lacking FBS and suspended in an equal volume of Matrigel membrane matrix in a final volume of 100 µL. Cells were injected subcutaneously into the right hind flank of CD-1 mice. Xenografts were used when they reached 150 – 300 mm^3^, median time of 33 (21 - 50) days (P3X63Ag8.653), and 34 (34 - 34) days for A-431 xenografts.

*Fluorescent imaging and biodistribution*

Three equivalently sized regions of interest (ROI) were drawn for each organ (xenografts, liver, kidneys, contralateral side, and background) and the mean corrected for number of pixels, signal in the contralateral, and the labelling ratio as follows:

$$Fluorescence\left( AU \right)=\frac{Signal_{Organ}-Signal_{Contralateral}}{LabellingRatio\times Area}$$

TBR was calculated as follows: $TBR=\frac{Signal_{Organ}}{Signal_{Contralteral}}$

Quantification of organs for biodistribution was the same as for the other images except they were not corrected for the contralateral signal.

P-values are calculated using ordinary two-way ANOVA with Sidak’s multiple comparison test, ns (p > 0.05), * (p <= 0.05), ** (p <= 0.01), ***(p <= 0.001), ****(p <= 0.0001).

*Pharmacokinetics*

Group 1: 10 min, and 1 hour, group 2: 5 min and 45 min, group 3: 15 min and 2 hours, blood was extracted from all groups at 3, 6, 24, 48, 72, 168, and 240 hrs. Samples were analyzed immediately to prevent clotting. The fluorescence per unit area in each capillary tube were analyzed by LICOR Odyssey in Image Studio (version 3.1). A standard curve was used to convert fluorescence to concentration.

*Acute and Delayed Toxicity*

Blood was collected while mice were anesthetized prior to euthanization via cardiac puncture for hematology and clinical chemistry. Blood (50 µL) was collected in a K2 EDTA coated tube for hematology. K2 EDTA coated tubes were made by adding 2.5 µL of a 50 mM stock solution of K2 EDTA. Tubes were dried in a biosafety cabinet overnight. A complete blood count was done on the whole blood samples. A minimum of 500 µL of blood was collected for clinical chemistry analysis into Microvette® 500 LH heparin-coated tubes (Sarstedt AG & Co). Blood was inverted 8 times and centrifuged at 1500 x g for 10 minutes. Plasma was collected and sent to Prairie Diagnostic Services (Saskatoon, SK) for clinical chemistry analysis. All animals were included in the analysis. No controlled blinding was implemented. Some values for the CBC parameters were flagged by the AcT Diff Hematology Analyzer were not available and were not included in the analysis. Clinical Chemistry tests were prioritized based on the volume required for the test. For some animals there was insufficient blood to perform the test. There is no data point available for these animals.

Organs/tissues (kidneys, spleen, liver, bone, heart, lungs, brain, skin, muscle, and testes/uterus) were inspected, collected, and stored in 10% neutral buffered formalin. The liver, spleen, and kidney were weighed.

Mice (n=48) were divided into two cohorts (Supplementary Figure 5), an acute toxicity cohort (sacrificed at day 2) and delayed toxicity cohort (sacrificed at day 14). Each cohort contained a female and male group comprised of twelve mice, untreated (n=2), vehicle control (n=5), and 14F7hT-IRDye800CW probe treated (n=5). Toxicity was monitored by complete blood counts (CBC), change in body weight, organ weight (liver, kidney, spleen), and a clinical chemistry panel measured at day 2 and day 14.

Statistical differences were observed for females at day 2 between vehicle and treatment groups for anion Gap (p = 0.023), and phosphorus (p = 0.0455), and between baseline and treatment groups for chloride (p = 0.0359) and urea (p = 0.0165). Males had statistical differences between baseline and treatment groups at day 2 for total protein (p = 0.429) and GLDH (p = 0.0437), and statistical differences at 14 days between vehicle and treatment for globulin (p = 0.004) and total protein (0.0272). No significant differences were observed for any parameters between both baseline vs treatment and vehicle vs treatment for any parameter.

Hematology parameters analyzed are listed in Supplementary Table 5 and results are provided in Supplementary Table 6 and Supplementary Table 7 (see also Supplementary Figure 9). There were no significant differences between both treatment vs vehicle and treatment vs baseline groups for any of the parameters. In males at 2 days there was a significant difference in HGB between baseline (145 ± 8) and treatment (158 ± 5) groups (p=0.0121). There was also a significant difference in RBC between baseline (8 ± 1) and treatment (10 ± 1) groups (p=0.0162). There were no significant differences between vehicle and treatment groups and no other statistical differences.

*Aggregate analysis of IHC studies using the 14F7 antibody*

Studies that included 14F7 immunohistochemistry (IHC) data and were accessible were manually curated. The following studies identified by pubmed identification (PMID) were included in the analysis (<PMID:16322892>, <PMID:19435393>, <PMID:21941577>, <PMID:21991524>, <PMID:22363862>, <PMID:22482082>, <PMID:22574836>, <PMID:24381785>, <PMID:24639871>, <PMID:25403557>, <PMID:26317019>, <PMID:26634172>, <PMID:31182063>). Analysis of aggregate data from these 13 studies shows 14F7 staining in 81.2% (580/714) of tumors and 4.9% (8/164) in normal tissues.

**Supplementary Figure 1.** Stability of 14F7hT-IRDye800CW probe*.* Evaluation of stability parameters of 14F7hT-IRDye800CW every 3 months. Dotted lines indicate proposed specifications. Linear fit is indicated by the black line, shaded area indicates 95% CI. Expiry is estimated where 95% CI intersects specification as per ICH Q1A(R2) and Q1E.


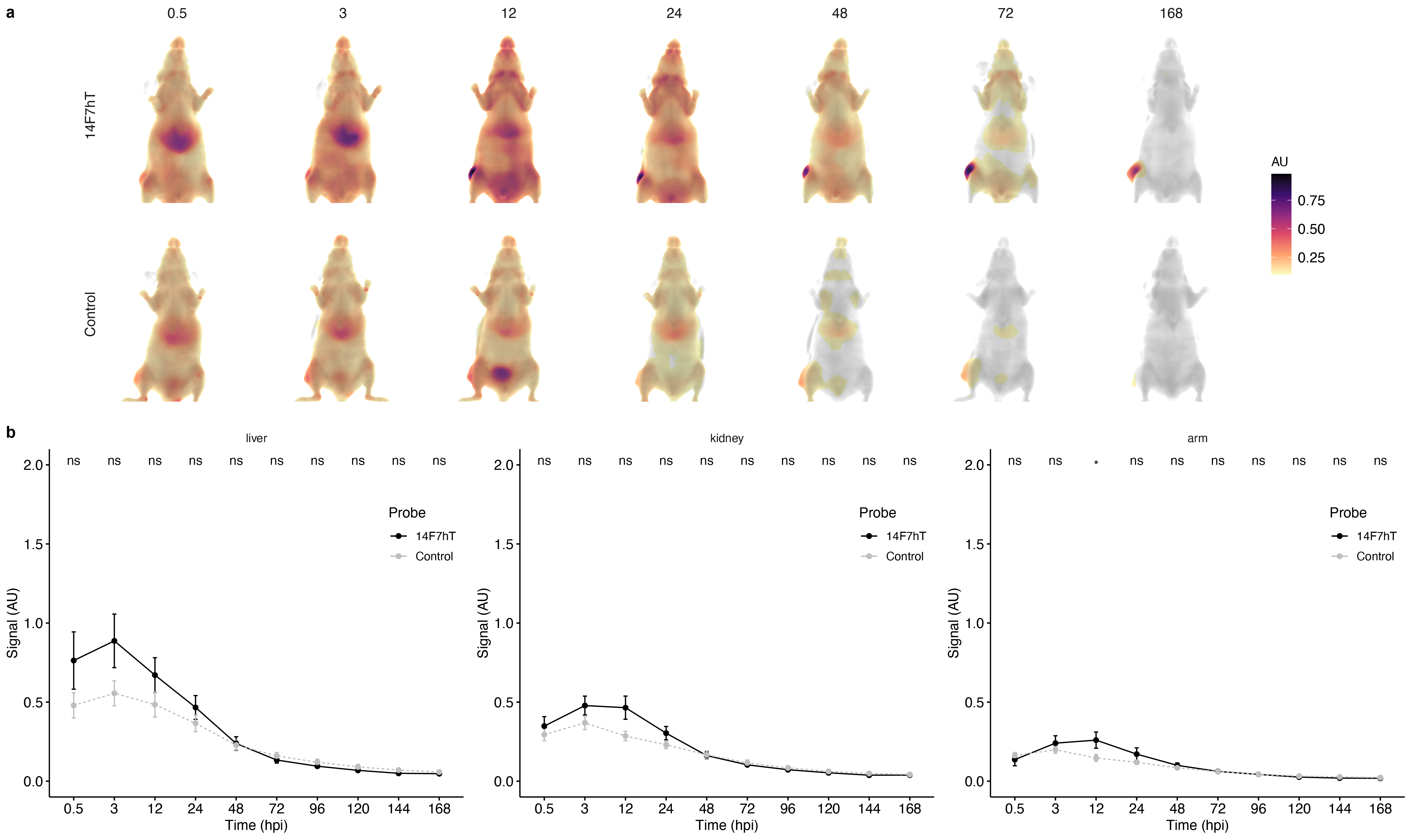


**Supplementary Figure 2.** Ventral analysis of 14F7hT-IRDye800CW versus control in mice bearing P3X63Ag8.653 xenografts. **(a)** Ventral fluorescent images of mice treated with 14F7hT-IRDye800CW or a control IgG-IRDye800CW at indicated time-points (hpi). (B) Quantification analysis of images for liver, kidney, and forearm (background). Signal is reported as MFI per pixel and is not corrected for contralateral fluorescence. Data is presented as mean ± sem, p-values calculated using ordinary two-way ANOVA with Sidak’s multiple comparisons test. ns (p > 0.05), * (p <= 0.05), ** (p <= 0.01), ***(p <= 0.001),*** *(p <= 0.0001).


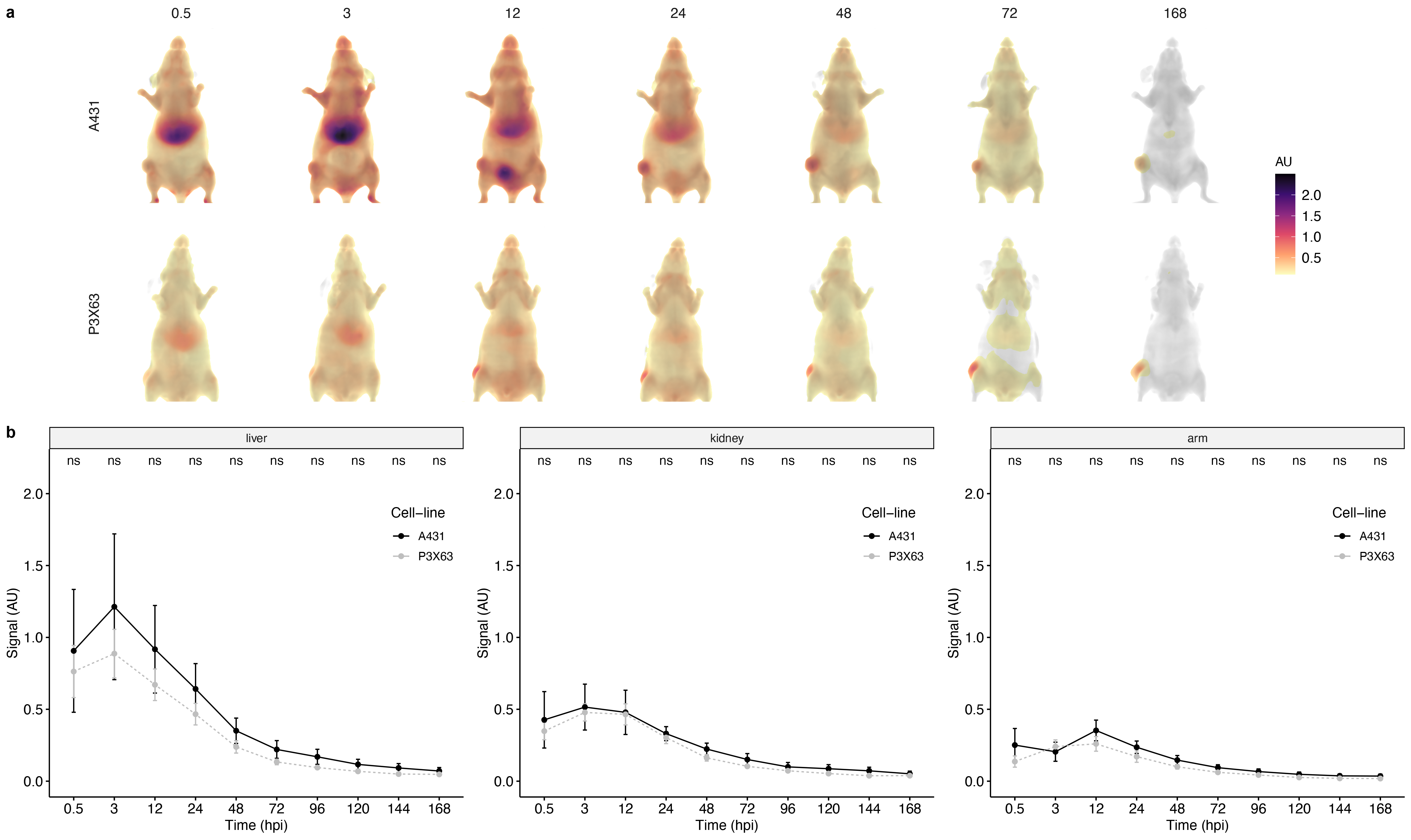


**Supplementary Figure 3.** Ventral images of 14F7hT-IRDye800CW injected into mice bearing Neu5GC-GM3-negative and -positive xenografts. **(a)** Ventral images of mice bearing Neu5GC-GM3-negative (A-431) and Neu5GC-GM3-positive (P3X63) xenografts. Time is indicated as hours post injection (hpi). **(b)** Quantification of the fluorescence in the liver, kidney, and arm in mouse images. Fluorescence is reported as MFI per pixel contralateral was not subtracted. Data is presented as mean ± sem, statistics are ordinary two-way anova using Sidak’s multiple comparison test. P3X63 = P3X63Ag8.653, A431 = A-431.


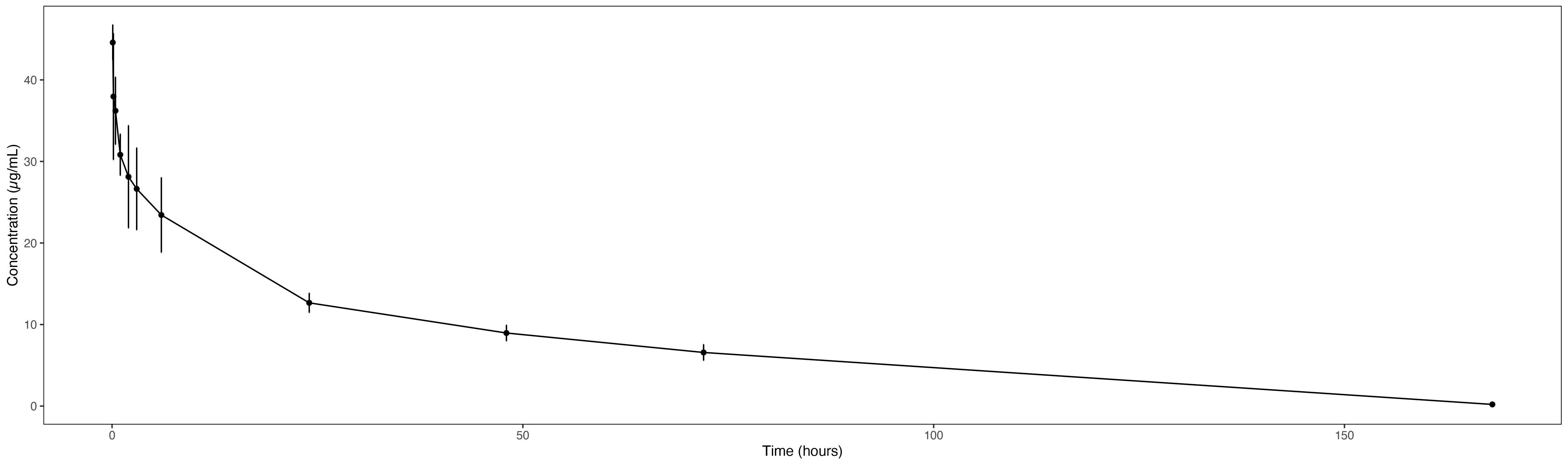


**Supplementary Figure 4.** Pharmacokinetic analysis. Female Balb/c mice (n=9), three per group were injected with a bolus dose of 0.5 nmoles (75 µg) 14F7hT-IRDye800CW via the tail vein. Blood was collected at 5 (n=3), 10 (n=3), 15 (n=3), 45 (n=3) min, 1 (n=3), 2 (n=3), 3 (n=9), 6 (n=9), 24 (n=9), 48 (n=9), 72 (n=9), 168 (n=9), and 240 (n=9) hpi. Fluorescence was converted to concentration using a standard curve.


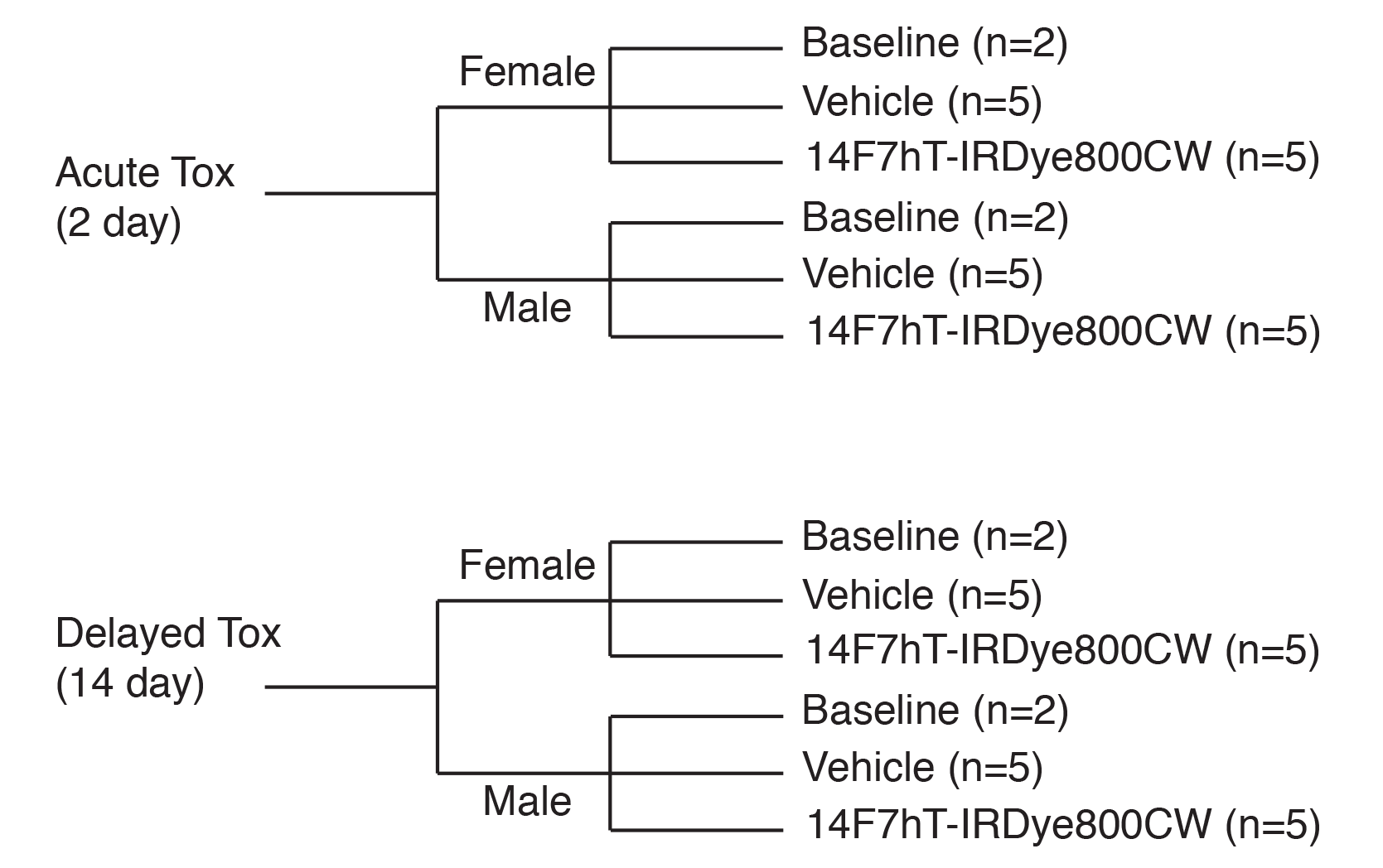


**Supplementary Figure 5**. Overview of toxicity study**.** Number of mice and collection time for acute (2-day) and delayed (14-day) single dose toxicity study. At collection CBC and clinical chemistry parameters were measured, followed by necroscopy.


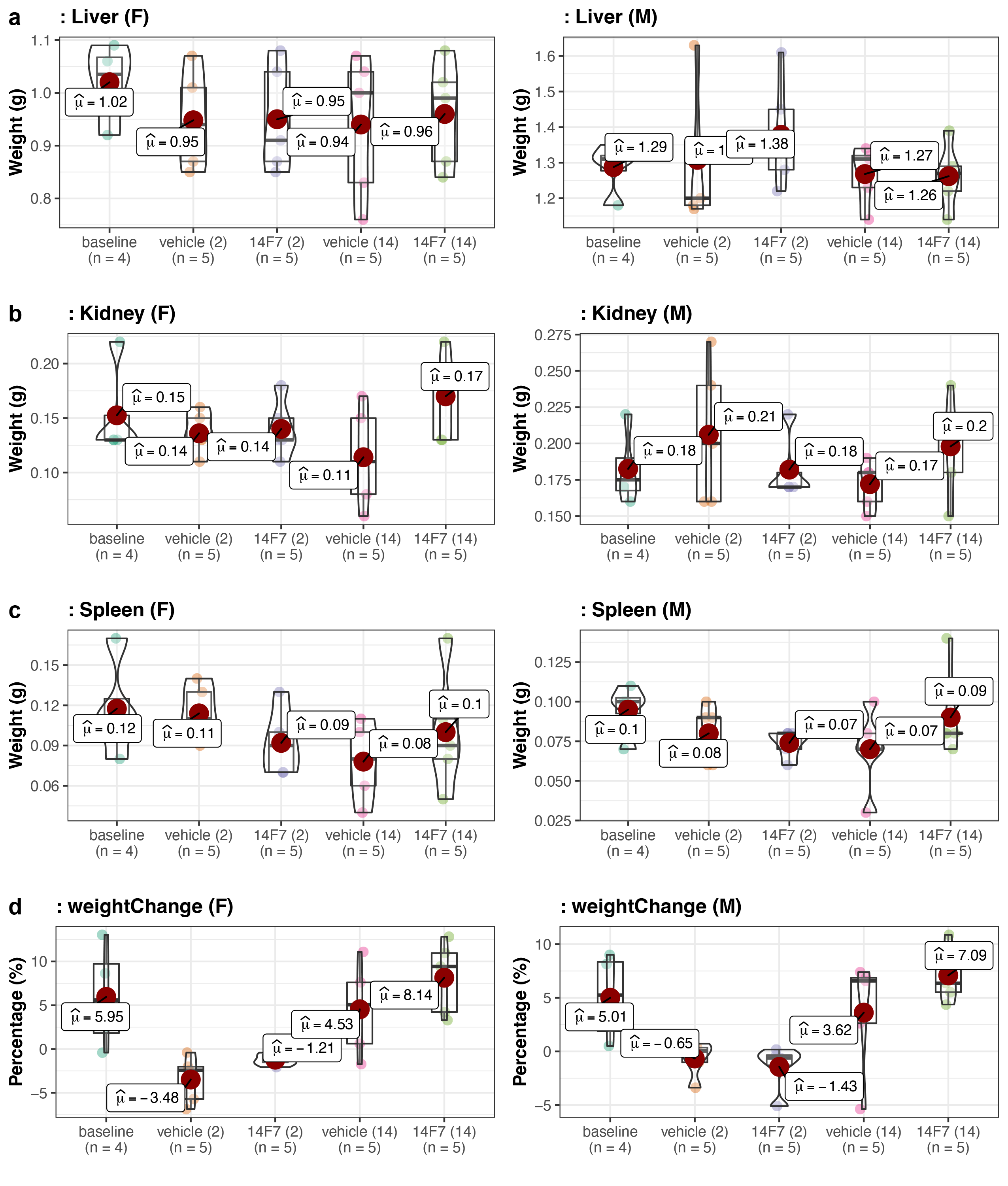


**Supplementary Figure 6.** Changes in organ and body weight during toxicity study*.* Violin plots comparing organ weights under different treatments. **(a)** Liver, **(b)** Kidney, **(c)** Spleen, and **(d)** Percent change in body weight. Day of sacrifice is shown in brackets, acute (2 days) and delayed (14 days). Baseline is untreated, vehicle is PBS, and 14F7 is 14F7hT-IRDye800CW probe. The number of animals in each group is indicated by (n) and female and male mice represented by (F) and (M), respectively. No significant differences between treatment and controls were observed.


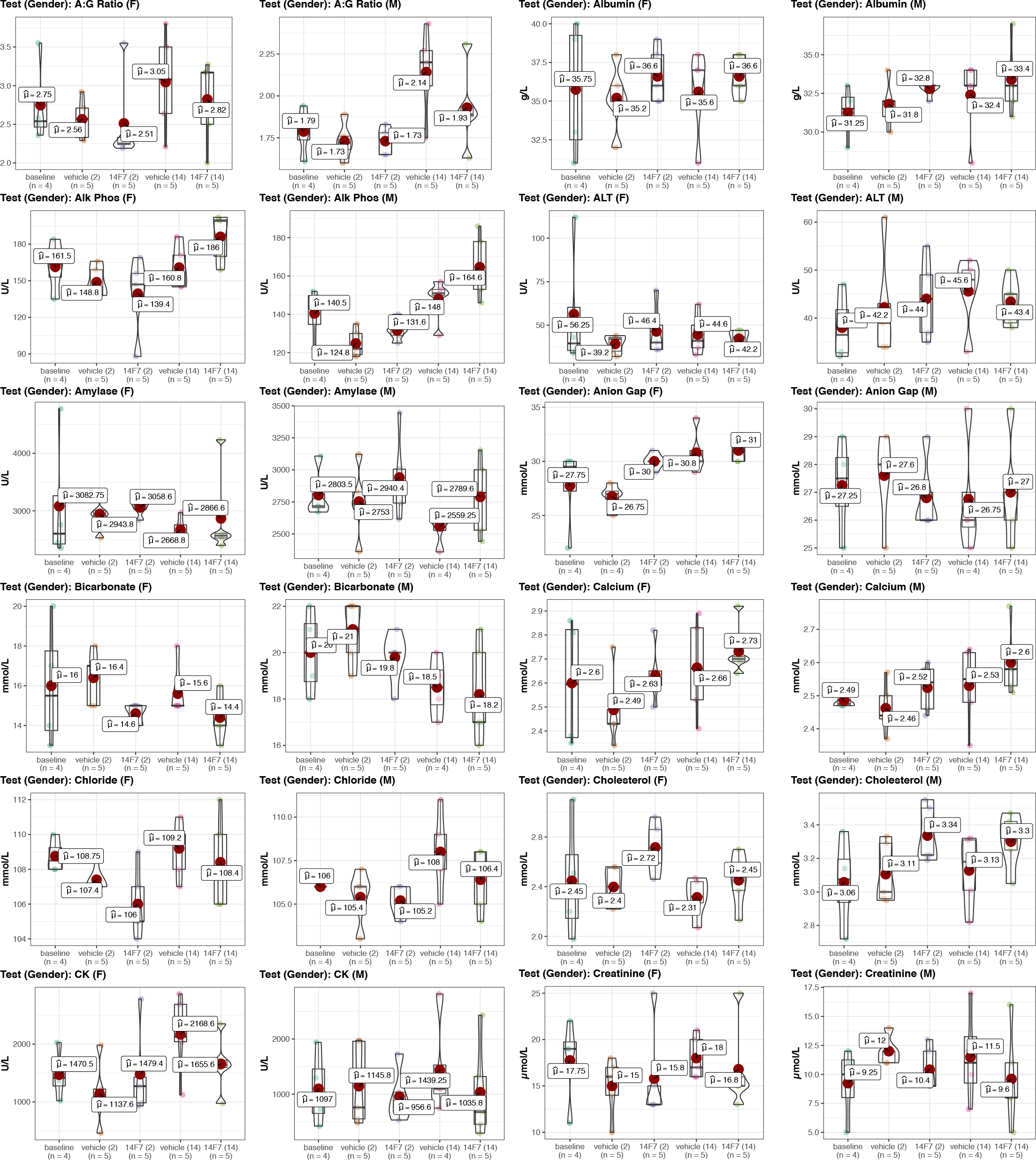


**Supplementary Figure 7.** | Clinical Chemistry (part 1/2). Graphical results of the following clinical chemistry parameters: Albumin/globulin ratio (A:G ratio), Albumin, Alkaline Phosphatse (Alk Phos); Alanine transaminase (ALT); Amylase, Anion Gap, Bicarbonate, Calcium, Chloride, Cholesterol, Creatine Kinase (CK), Creatine. M = male, F = Female, n = number of mice. Study day is indicated in parentheses.


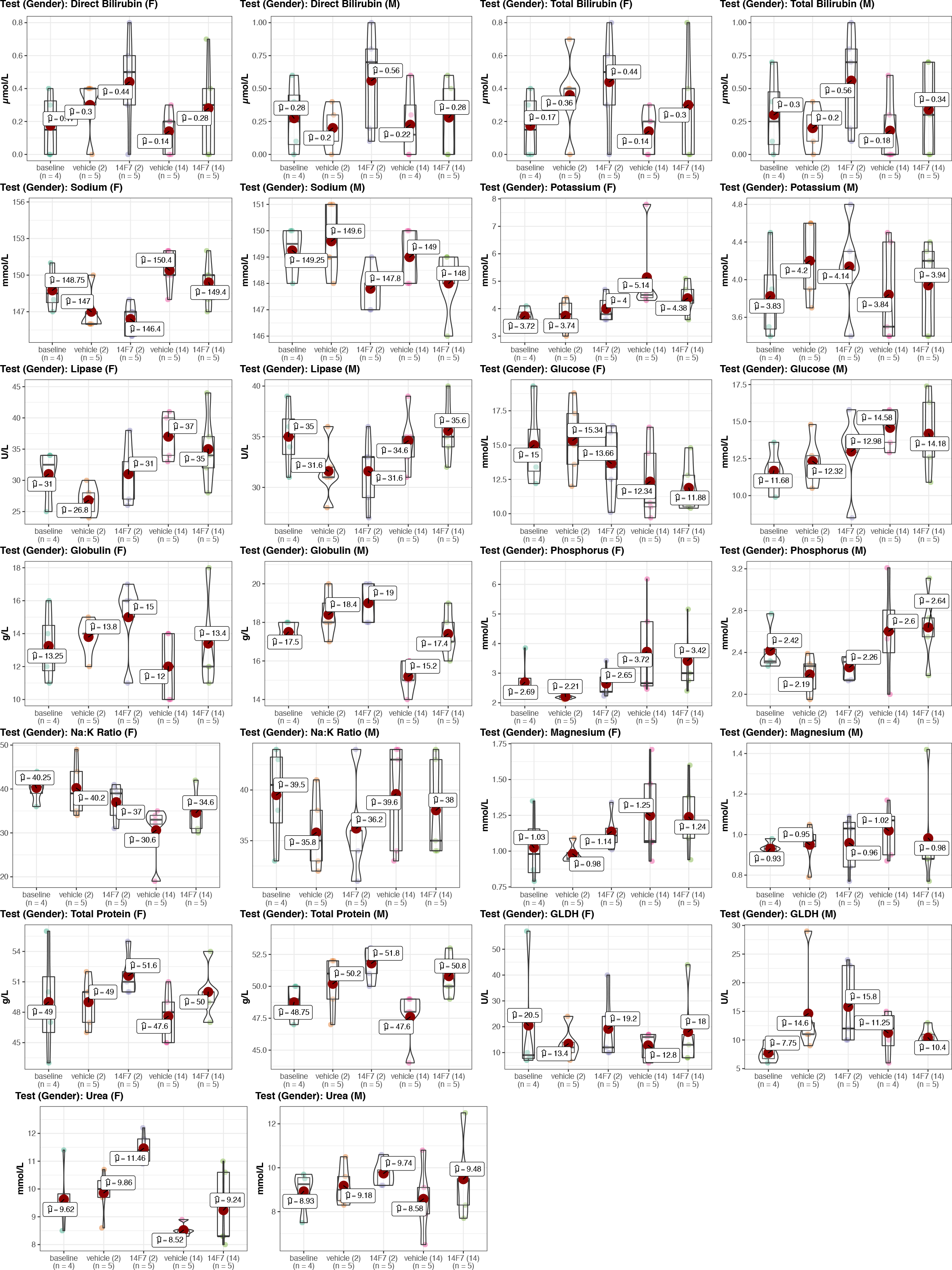


**Supplementary Figure 8.** Clinical Chemistry (part 2/2). Graphical results of the following clinical chemistry parameters: Direct Bilirubin, Total Bilirubin, Sodium, Potassium, Lipase, Glucose, Globulin, Phosphorus, Sodium-potassium (Na:K ratio), Magnesium, Total protein, Glutamate dehydrogenase (GLDH), Urea. M = male, F = Female, n = number of mice. Study day is indicated in parentheses.


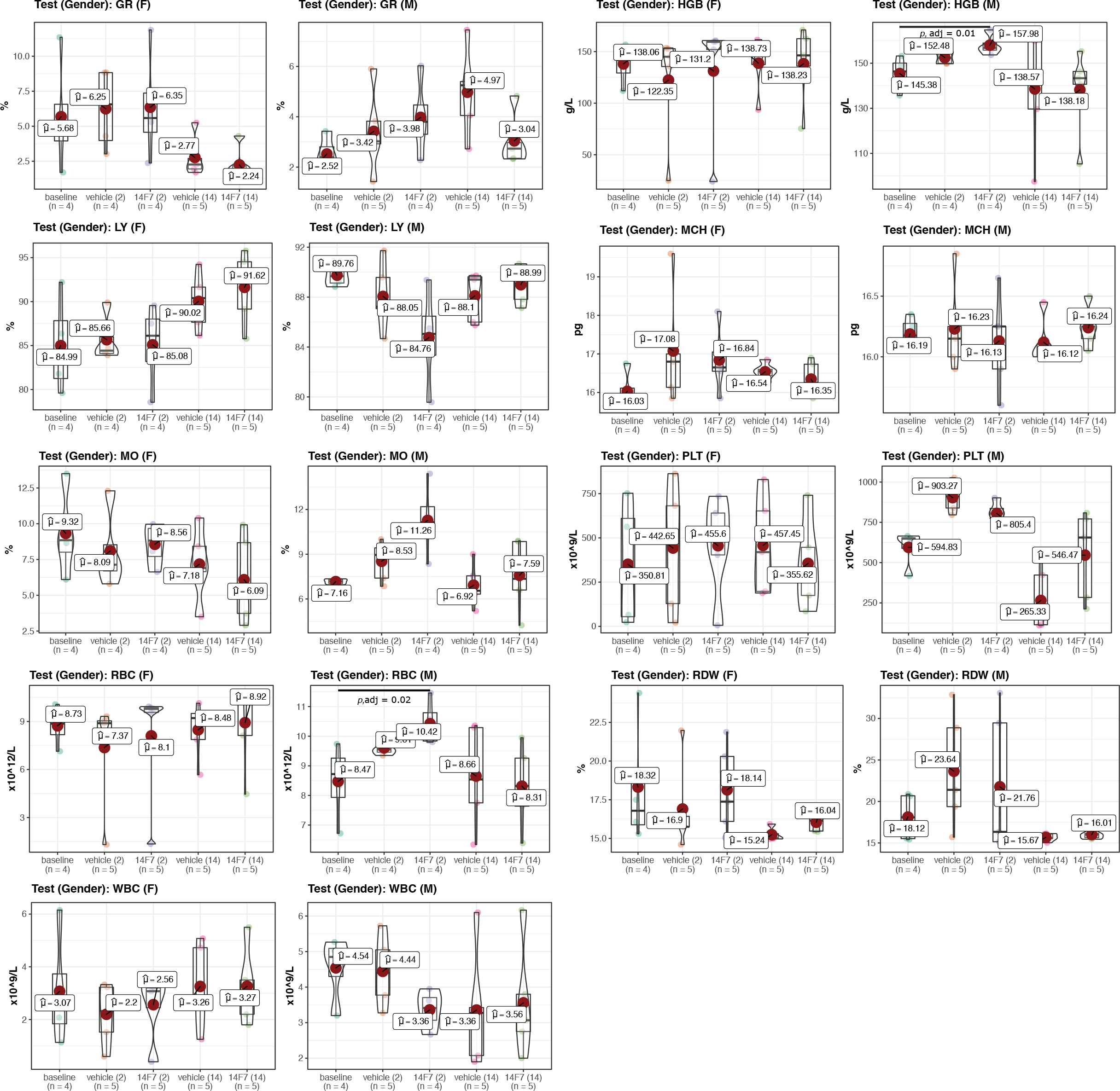


**Supplementary Figure 9.** Complete blood counts (CBC). Graphical results of the following CBC parameters: Granulocytes (GR), Hemoglobin (HGB), Lymphocytes (LY), Mean cell hemoglobin (MCH), Monocytes (MO), Platelet count (PLT), Red blood cell count (RBC), Red blood cell distribution width (RDW), White blood cell count (WBC). M= male, F= Female, n = number of mice. Study day is indicated in parentheses.

*Tables*

Supplementary Table 1. Release specifications for 14F7hT-IRDye800CW probe

| Test | Method | Method code | Specification |
| --- | --- | --- | --- |
| Visual appearance | Visual | In-house | Clear green liquid free of any visible particulates |
| Strength | UV | In-house | 5 ± 1 mg/mL |
| Labelling ratio (Dye:Protein) | UV | In-house | 0.7 - 2.0 |
| Purity (fluorescence) | SDS-PAGE/NIR | In-house | >= 90% |
| Purity (protein) | Micro-capillary electrophoresis | In-house | >= 95% |
| Molecular weight (kDa) | Micro-capillary electrophoresis | In-house | 150 ± 10% kDa |
|  |  |  |  |
| Functional assay (binding) | Flow cytometry |  | Binding constant of sample within 2-fold of reference |
| pH | pH meter | USP <791> | 6.5 - 7.5 |
| Endotoxin | LAL | USP <85> | <= 16 EU/mL |

Supplementary Table 2. Clinical chemistry parameters measured

| Abbreviation | Parameter | Units |
| --- | --- | --- |
| A:G Ratio | Albumin-globulin |  |
| ALB | Albumin | g/L |
| ALP | Alkaline phosphatase | U/L |
| ALT | Alanine transaminase | U/L |
| Amy | Amylase | U/L |
| Anion Gap | Anion Gap | mmol/L |
| TCO2 | Bicarbonate | mmol/L |
| Ca | Calcium | mmol/L |
| Cl | Chloride | mmol/L |
| Cholesterol | Cholesterol | mmol/L |
| CK | Creatine Kinase | U/L |
| Creat | Creatinine | µmol/L |
| Direct Bilirubin | Direct Bilirubin | µmol/L |
| GGT | Gamma-glutamyl transpeptidase | U/L |
| GLDH | Glutamate dehydrogenase | U/L |
| Globulin | Globulin | g/L |
| Glucose | Glucose | mmol/L |
| Indirect Bilirubin | Indirect Bilirubin | µmol/L |

Supplementary Table 3. Clinical chemistry results for male mice in toxicity study

| **Test** | | **Males** | | | | |
| --- | --- | --- | --- | --- | --- | --- |
|  | | Acute (mean ± SD) | | Delayed (mean ± SD) | | (mean ± SD) |
| Clinical Chemistry | Units | vehicle | 14F7 | vehicle | 14F7 | baseline |
| A:G Ratio |  | 1.7 ± 0.1 | 1.7 ± 0.1 | 2.1 ± 0.3 | 1.9 ± 0.2 | 1.7 ± 0.2 |
| ALT | U/L | 40 ± 10 | 44 ± 8 | 46 ± 8 | 43 ± 5 | 31 ± 9 |
| Albumin | g/L | 32 ± 2 | 32.8 ± 0.4 | 32 ± 3 | 33 ± 2 | 30 ± 2 |
| Alk Phos | U/L | 125 ± 8 | 132 ± 6 | 150 ± 10 | 160 ± 20 | 130 ± 20 |
| Amylase | U/L | 2800 ± 300 | 2900 ± 300 | 2600 ± 100 | 2800 ± 300 | 2800 ± 600 |
| Anion Gap | mmol/L | 28 ± 2 | 27 ± 1 | 27 ± 2 | 27 ± 2 | 32 ± 6 |
| Bicarbonate | mmol/L | 21 ± 1 | 20 ± 1 | 19 ± 1 | 18 ± 2 | 17 ± 4 |
| CK | U/L | 1100 ± 800 | 1000 ± 500 | 1400 ± 900 | 1000 ± 900 | 700 ± 600 |
| Calcium | mmol/L | 2.5 ± 0.1 | 2.5 ± 0.1 | 2.5 ± 0.1 | 2.6 ± 0.1 | 2.4 ± 0.2 |
| Chloride | mmol/L | 105 ± 2 | 105.2 ± 0.8 | 108 ± 2 | 106 ± 2 | 105 ± 1 |
| Cholesterol | mmol/L | 3.1 ± 0.2 | 3.3 ± 0.2 | 3.1 ± 0.2 | 3.3 ± 0.2 | 3.2 ± 0.2 |
| Creatinine | µmol/L | 12 ± 1 | 10 ± 2 | 12 ± 4 | 10 ± 4 | 13 ± 5 |
| Direct Bilirubin | µmol/L | 0.2 ± 0.2 | 0.6 ± 0.4 | 0.2 ± 0.3 | 0.3 ± 0.3 | 0.1 ± 0.2 |
| GGT | U/L | 0 ± 0 | 0 ± 0 | 0 ± 0 | 0 ± 0 | 0 ± 0 |
| GLDH | U/L | 15 ± 8 | 16 ± 7 | 11 ± 4 | 10 ± 2 | 8 ± 1 |
| Globulin | g/L | 18 ± 1 | 19 ± 1 | 15.2 ± 0.8 | 17 ± 1 | 18 ± 1 |
| Glucose | mmol/L | 12 ± 2 | 13 ± 3 | 15 ± 1 | 14 ± 3 | 14 ± 3 |
| Indirect Bilirubin | µmol/L | 0 ± 0 | 0 ± 0 | 0 ± 0 | 0.1 ± 0.1 | 0.1 ± 0.1 |
| Lipase | U/L | 32 ± 3 | 32 ± 4 | 35 ± 3 | 36 ± 3 | 31 ± 5 |
| Magnesium | mmol/L | 0.9 ± 0.1 | 1.0 ± 0.1 | 1.0 ± 0.1 | 1.0 ± 0.3 | 1.0 ± 0.1 |
| Na:K Ratio |  | 36 ± 4 | 36 ± 5 | 40 ± 6 | 38 ± 5 | 38 ± 4 |
| Phosphorus | mmol/L | 2.2 ± 0.2 | 2.3 ± 0.1 | 2.6 ± 0.5 | 2.6 ± 0.3 | 2.6 ± 0.3 |
| Potassium | mmol/L | 4.2 ± 0.4 | 4.1 ± 0.5 | 3.8 ± 0.6 | 3.9 ± 0.5 | 4 ± 0.4 |
| Sodium | mmol/L | 150 ± 1 | 147.8 ± 0.8 | 149 ± 1 | 148 ± 1 | 150 ± 2 |
| Total Bilirubin | µmol/L | 0.2 ± 0.2 | 0.6 ± 0.4 | 0.2 ± 0.3 | 0.3 ± 0.4 | 0.2 ± 0.3 |
| Total Protein | g/L | 50 ± 2 | 52 ± 1 | 48 ± 2 | 51 ± 2 | 48 ± 1 |
| Urea | mmol/L | 9.2 ± 0.9 | 9.7 ± 0.6 | 9 ± 2 | 10 ± 2 | 8.8 ± 0.7 |

Supplementary Table 4. Clinical chemistry results for female mice in toxicity study

| **Test** | | **Females** | | | | |
| --- | --- | --- | --- | --- | --- | --- |
|  | | Acute (mean ± SD) | | Delayed (mean ± SD) | | (mean ± SD) |
| Clinical Chemistry | Units | vehicle | 14F7 | vehicle | 14F7 | baseline |
| A:G Ratio |  | 2.6 ± 0.3 | 2.5 ± 0.6 | 3 ± 0.6 | 2.8 ± 0.6 | 2.4 ± 0.5 |
| ALT | U/L | 39 ± 5 | 50 ± 10 | 40 ± 10 | 42 ± 5 | 40 ± 30 |
| Albumin | g/L | 35 ± 2 | 37 ± 2 | 36 ± 3 | 37 ± 1 | 34 ± 4 |
| Alk Phos | U/L | 150 ± 10 | 140 ± 30 | 160 ± 20 | 190 ± 20 | 140 ± 30 |
| Amylase | U/L | 2900 ± 300 | 3100 ± 100 | 2700 ± 200 | 2900 ± 800 | 2700 ± 900 |
| Anion Gap | mmol/L | 27 ± 1 | 30.0 ± 0.7 | 31 ± 2 | 31 ± 1 | 32 ± 6 |
| Bicarbonate | mmol/L | 16 ± 1 | 14.6 ± 0.5 | 16 ± 1 | 14 ± 1 | 14 ± 4 |
| CK | U/L | 1100 ± 500 | 1500 ± 800 | 2200 ± 700 | 1700 ± 500 | 900 ± 600 |
| Calcium | mmol/L | 2.5 ± 0.2 | 2.6 ± 0.1 | 2.7 ± 0.2 | 2.7 ± 0.1 | 2.5 ± 0.2 |
| Chloride | mmol/L | 107.4 ± 0.9 | 106 ± 2 | 109 ± 2 | 108 ± 3 | 107 ± 2 |
| Cholesterol | mmol/L | 2.4 ± 0.2 | 2.7 ± 0.2 | 2.3 ± 0.2 | 2.5 ± 0.2 | 2.4 ± 0.3 |
| Creatinine | µmol/L | 15 ± 3 | 16 ± 5 | 18 ± 2 | 17 ± 5 | 18 ± 4 |
| Direct Bilirubin | µmol/L | 0.3 ± 0.2 | 0.4 ± 0.3 | 0.1 ± 0.1 | 0.3 ± 0.3 | 0.2 ± 0.2 |
| GGT | U/L | 0 ± 0 | 0 ± 0 | 0 ± 0 | 0 ± 0 | 0 ± 0 |
| GLDH | U/L | 13 ± 7 | 20 ± 10 | 13 ± 5 | 18 ± 15 | 15 ± 17 |
| Globulin | g/L | 14 ± 1 | 15 ± 2 | 12 ± 2 | 13 ± 3 | 15 ± 2 |
| Glucose | mmol/L | 15 ± 3 | 14 ± 3 | 12 ± 3 | 12 ± 2 | 15 ± 2 |
| Indirect Bilirubin | µmol/L | 0.1 ± 0.1 | 0 ± 0 | 0 ± 0 | 0 ± 0 | 0.1 ± 0.2 |
| Lipase | U/L | 27 ± 2 | 31 ± 5 | 37 ± 4 | 35 ± 6 | 29 ± 5 |
| Magnesium | mmol/L | 1 ± 0.1 | 1.1 ± 0.1 | 1.2 ± 0.3 | 1.2 ± 0.3 | 1.1 ± 0.2 |
| Na:K Ratio |  | 40 ± 6 | 37 ± 4 | 31 ± 7 | 35 ± 5 | 40 ± 2 |
| Phosphorus | mmol/L | 2.2 ± 0.1 | 2.6 ± 0.5 | 4 ± 2 | 3 ± 1 | 3.0 ± 0.6 |
| Potassium | mmol/L | 3.7 ± 0.6 | 4.0 ± 0.5 | 5.1 ± 1.5 | 4.4 ± 0.6 | 3.7 ± 0.2 |
| Sodium | mmol/L | 147 ± 2 | 146 ± 1 | 150 ± 2 | 149 ± 2 | 149 ± 1 |
| Total Bilirubin | µmol/L | 0.4 ± 0.3 | 0.4 ± 0.3 | 0.1 ± 0.1 | 0.3 ± 0.3 | 0.3 ± 0.2 |
| Total Protein | g/L | 49 ± 2 | 52 ± 2 | 48 ± 3 | 50 ± 3 | 49 ± 4 |
| Urea | mmol/L | 9.9 ± 0.8 | 11.5 ± 0.5 | 8.5 ± 0.2 | 9 ± 1 | 10 ± 1 |

Table 5. CBC parameters

| Abbreviation | Parameter | Units |
| --- | --- | --- |
| WBC | White blood cell count | 109/L |
| PLT | Platelet count | 109/L |
| RBC | Red blood cell count | 1012/L |
| RDW | Red blood cell distribution width | % |
| GR | Granulocytes | % |
| MO | Monocytes | % |
| LY | Lymphocytes | % |
| HGB | Hemoglobin | g/L |
| MCH | Mean cell hemoglobin | pg |

Table 6. CBC values for male mice in toxicity study

| **Test** | | **Males** | | | | |
| --- | --- | --- | --- | --- | --- | --- |
|  |  | Acute (mean ± SD) |  | Delayed (mean ± SD) |  |  |
| CBC | Units | vehicle | 14F7 | vehicle | 14F7 | baseline |
| GR | % | 3 ± 2 | 4 ± 2 | 5 ± 2 | 3 ± 1 | 2.5 ± 0.7 |
| HCT | L/L | 0.5 ± 0 | 0.5 ± 0 | 0.4 ± 0.1 | 0.4 ± 0.1 | 0.5 ± 0 |
| HGB | g/L | 152 ± 3 | 158 ± 5 | 140 ± 30 | 140 ± 20 | 145 ± 8 |
| LY | % | 88 ± 3 | 85 ± 4 | 88 ± 2 | 89 ± 2 | 89.8 ± 0.9 |
| MCH | pg | 16.2 ± 0.4 | 16.1 ± 0.4 | 16.1 ± 0.2 | 16.2 ± 0.2 | 16.2 ± 0.1 |
| MCHC | g/L | 321.6 ± 0.1 | 321.8 ± 0.2 | 321 ± 1 | 321 ± 1 | 321.3 ± 0.3 |
| MCV | fl | 50 ± 1 | 47 ± 2 | 50 ± 3 | 52 ± 4 | 54 ± 6 |
| MO | % | 9 ± 1 | 11 ± 3 | 7 ± 1 | 8 ± 2 | 7.2 ± 0.2 |
| PLT | x10^9/L | 900 ± 90 | 800 ± 80 | 300 ± 200 | 500 ± 300 | 600 ± 100 |
| RBC | x10^12/L | 9.6 ± 0.3 | 10.4 ± 0.8 | 9 ± 2 | 8 ± 1 | 9 ± 1 |
| RDW | % | 24 ± 7 | 22 ± 9 | 15.7 ± 0.5 | 16 ± 0.5 | 18 ± 3 |
| WBC | x10^9/L | 4 ± 1 | 3.4 ± 0.6 | 3 ± 2 | 4 ± 2 | 4.5 ± 0.9 |

Table 7. CBC values for female mice in toxicity study

| **Test** | | **Females** | | | | |
| --- | --- | --- | --- | --- | --- | --- |
|  |  | Acute (mean ± SD) |  | Delayed (mean ± SD) |  |  |
| CBC | Units | vehicle | 14F7 | vehicle | 14F7 | baseline |
| GR | % | 6 ± 3 | 6 ± 4 | 3 ± 1 | 2 ± 1 | 6 ± 4 |
| HCT | L/L | 0.4 ± 0.2 | 0.4 ± 0.2 | 0.4 ± 0.1 | 0.4 ± 0.1 | 0.4 ± 0.1 |
| HGB | g/L | 120 ± 50 | 130 ± 60 | 140 ± 30 | 140 ± 40 | 140 ± 20 |
| LY | % | 86 ± 3 | 85 ± 5 | 90 ± 3 | 92 ± 4 | 85 ± 6 |
| MCH | pg | 17 ± 2 | 16.8 ± 0.8 | 16.5 ± 0.2 | 16.3 ± 0.5 | 16 ± 0.5 |
| MCHC | g/L | 320 ± 10 | 320 ± 10 | 321 ± 2 | 321 ± 3 | 321 ± 1 |
| MCV | fl | 54 ± 6 | 52 ± 5 | 51 ± 2 | 50 ± 4 | 49.3 ± 0.8 |
| MO | % | 8 ± 3 | 9 ± 2 | 7 ± 3 | 6 ± 3 | 9 ± 3 |
| PLT | x10^9/L | 400 ± 400 | 500 ± 300 | 500 ± 300 | 400 ± 300 | 400 ± 400 |
| RBC | x10^12/L | 7 ± 3 | 8 ± 4 | 9 ± 2 | 9 ± 3 | 9 ± 1 |
| RDW | % | 17 ± 3 | 18 ± 3 | 15.2 ± 0.4 | 16 ± 0.6 | 18 ± 4 |
| WBC | x10^9/L | 2 ± 1 | 3 ± 1 | 3 ± 2 | 3 ± 1 | 3 ± 2 |
